# Near-infrared-IIb emitting single-atom catalyst for imaging-guided therapy of blood-brain barrier breakdown after traumatic brain injury

**DOI:** 10.1038/s41467-023-35868-8

**Published:** 2023-01-13

**Authors:** Biao Huang, Tao Tang, Shi-Hui Chen, Hao Li, Zhi-Jun Sun, Zhi-Lin Zhang, Mingxi Zhang, Ran Cui

**Affiliations:** 1 College of Chemistry and Molecular Sciences, Wuhan University, 430072 Wuhan, China; 2 State Key Laboratory of Advanced Technology for Materials Synthesis and Processing, Wuhan University of Technology, 430070 Wuhan, China; 3 The State Key Laboratory Breeding Base of Basic Science of Stomatology (Hubei-MOST) & Key Laboratory of Oral Biomedicine Ministry of Education, School & Hospital of Stomatology, Wuhan University, 430079 Wuhan, China

**Keywords:** Nanoparticles, Imaging techniques and agents, Brain injuries

## Abstract

The blood-brain barrier breakdown, as a prominent feature after traumatic brain injury, always triggers a cascade of biochemical events like inflammatory response and free radical-mediated oxidative damage, leading to neurological dysfunction. The dynamic monitoring the status of blood-brain barrier will provide potent guidance for adopting appropriate clinical intervention. Here, we engineer a near-infrared-IIb Ag_2_Te quantum dot-based Mn single-atom catalyst for imaging-guided therapy of blood-brain barrier breakdown of mice after traumatic brain injury. The dynamic change of blood-brain barrier, including the transient cerebral hypoperfusion and cerebrovascular damage, could be resolved with high spatiotemporal resolution (150 ms and ~ 9.6 µm). Notably, the isolated single Mn atoms on the surface of Ag_2_Te exhibited excellent catalytic activity for scavenging reactive oxygen species to alleviate neuroinflammation in brains. The timely injection of Mn single-atom catalyst guided by imaging significantly promoted the reconstruction of blood-brain barrier and recovery of neurological function after traumatic brain injury.

## Abstract (web summary)

Monitoring the status of blood-brain barrier (BBB) and inhibiting reactive oxygen species (ROS)-mediated oxidative damage are key issues in the treatment of traumatic brain injury (TBI). Here, the authors design a near-infrared-IIb emitting Mn single-atom catalyst for imaging-guided therapy to alleviate ROS mediated neuroinflammation in the brain and simultaneously obtain timely feedback of therapeutic effect, promoting the reconstruction of BBB and recovery of neurological function after TBI in mice.

## Introduction

Traumatic brain injury (TBI), as the devastating neurological disease^1^, has been listed as the main cause of death and disability among young adults, posing a major threat to global health^2^. The cerebral micro-vessels, as the principal part of blood-brain barrier (BBB)^3,4^, are vulnerable to external force damage, thus, the BBB impairment is a common and prominent pathological feature in TBI^5^. However, the cerebral microvascular rupture (smaller than 12 μm)^6^ and the cerebral hypoperfusion, as two key evidences of BBB breakdown^5,7^, are still difficult to visualize due to the limited spatiotemporal resolution of conventional imaging technologies^8,9^. In addition, following the primary injury caused by external force, the secondary BBB injury such as brain edema and nerve inflammation may cause long-term brain pathologies and lead to serious neurodegenerative diseases^10–12^. Early intervention and treatment of TBI complications become the key for the prevention of brain diseases^13^.

One of principle manifestations of secondary injury in TBI is nerve inflammation^5^, which is caused by the over-production of reactive oxygen species (ROS), such as superoxide radical (^•^O_2_^−^), hydroxyl radical (^•^OH), and hydrogen peroxide (H_2_O_2_)^11^. However, endogenous antioxidant enzymes are insufficient to remove excessive ROS^14,15^, so introducing the exogenous ROS scavengers is the critical strategy to alleviate ROS-mediated secondary injury and improve TBI treatment effect^16^. At present, several artificial nano-catalysts with multi-enzyme mimicking (superoxide dismutase and catalase) and stable antioxidant activity in vivo, have been successfully applied in the pre-clinical neuroprotective therapy for brain injury related diseases^17–23^. However, the present neuroprotective strategies are still difficult to provide timely feedback for the evaluation of therapeutic efficacy^17^. Quantum dots (QDs) emitting in the long-wavelength region of near-infrared-II (NIR-IIb, 1500–1700 nm) can provide the noninvasive in vivo imaging with high spatiotemporal resolution for the cerebral microvascular imaging^24–28^. More importantly, the appropriate elements doping can effectively provide catalytic ability to QDs^29–31^. Therefore, by constructing the NIR-IIb QDs-based single-atom catalyst (SAC) with antioxidant catalytic activity, the BBB repair processes could be monitored under the ROS scavenging therapy with dynamic NIR-IIb imaging for timely therapeutic effect evaluation, providing the potent guidance for accurate and more effective TBI treatment.

**Fig. 1:**
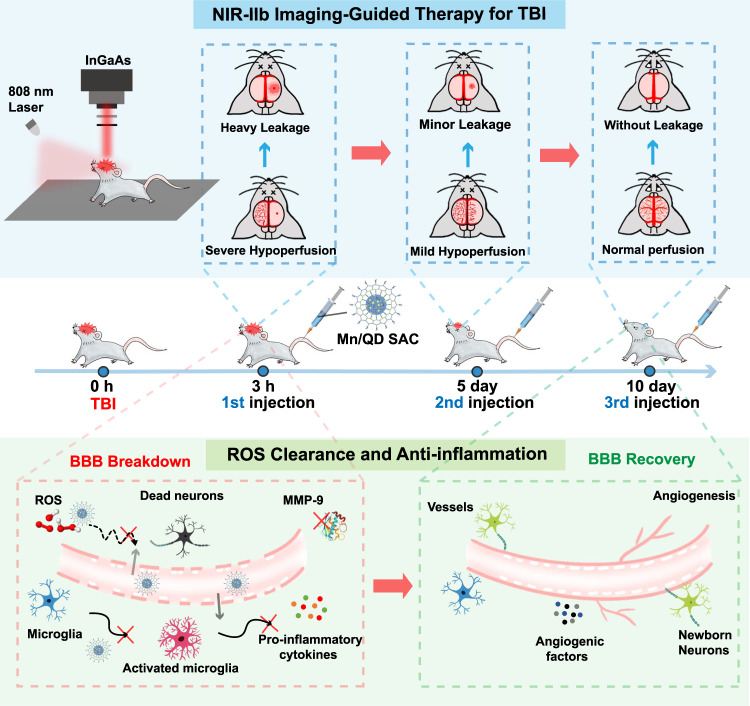
Schematic diagram of Mn/QD single-atom catalyst for imaging-guided TBI therapy. The near-infrared-IIb emitting Mn single-atom catalyst enables dynamically monitoring the BBB status in a noninvasive way and repressing the ROS-mediated BBB breakdown.

Mn is an essential element as the active center of various enzymes in organisms, which are closely related to redox, electron transfer and the Lewis acid catalysis processes in life activities^32^. Herein, we develop a Ag_2_Te QD-based Mn single-atom catalyst (Mn/QD SAC) emitting in the NIR-IIb window (at ~1690 nm) with multi-enzyme mimicking catalytic activities, which is a promising candidate for the imaging-guided therapy of damaged BBB after TBI (Fig. 1). Mn/QD SAC afforded high temporal (150 ms) and spatial (~9.6 µm) resolution for the accurate assessment of BBB status in real time. Furthermore, due to the atomically dispersed Mn on the surface of Ag_2_Te nanocrystals, Mn/QD SAC displayed catalase (CAT)-like and superoxide dismutase (SOD)-like antioxidant activity to effectively remove H_2_O_2_ (98%), ^•^O_2_^−^ (97%), and ^•^OH (81%) with high elimination rate, thus significantly reducing the intracellular ROS level (nearly 99%). Notably, the down-regulated expression of matrix metalloproteinase 9 (MMP-9) and pro-inflammatory cytokines in brains also verified the antioxidant effect of Mn/QD SAC. Collectively, the timely injection of Mn/QD SAC guided by NIR-IIb imaging significantly alleviated the ROS-mediated neuroinflammation in brains of mice after TBI, which further promoted the BBB reconstruction and neurological function recovery.

## Results

### Structural and optical characterizations of Mn/QD SAC

**Fig. 2:**
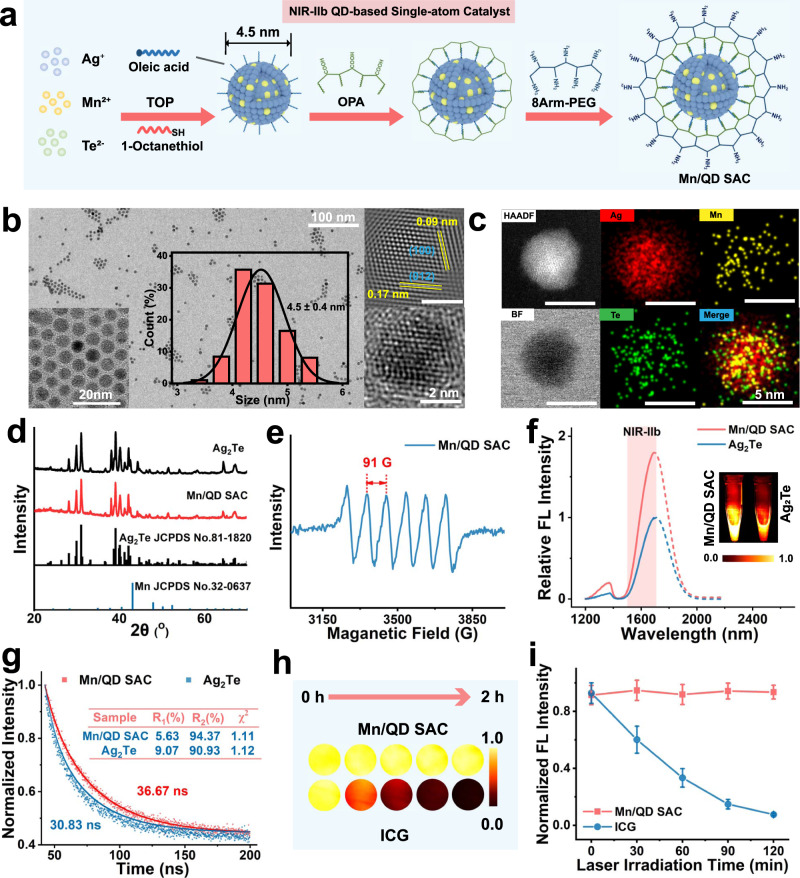
Preparation of NIR-IIb QD-based single-atom catalyst with antioxidant activity and fluorescence properties. **a** Schematic diagram of synthesis and construction of Mn/QD SAC. **b** The TEM image, high-resolution HAADF-STEM image, and size distribution statistics of Mn/QD SAC. Data are presented as means ± SD (*n* = 300 particles). **c** HAADF-STEM images and energy-dispersive X-ray spectroscopy (EDS) mapping of Mn/QD SAC. **d** XRD patterns of Mn/QD SAC and Ag_2_Te QDs. **e** The ESR pattern of Mn/QD SAC. **f** Fluorescence emission spectra of Mn/QD SAC and Ag_2_Te QDs. The inset images were the NIR-IIb images of Mn/QD SAC and Ag_2_Te QDs respectively. **g** Time-resolved photoluminescence decay curves of Mn/QD SAC and Ag_2_Te QDs. **h** Fluorescence images of Mn/QD SAC and ICG under the 808 nm laser irradiation, which was taken at different time points (0, 30, 60, 90, and 120 min). **i** Normalized fluorescence intensity changes of Mn/QD SAC and ICG verse time according to the fluorescence images in panel **h**. Data are presented as means ± SD (*n* = 3 independent samples). Source data are provided as a Source Data file.

Mn/QD SAC was constructed according to the steps in Fig. 2a. At present, only few types of QDs without toxic heavy metal can reach to NIR-IIb window^33,34^. Ag_2_Te QDs, with narrow bandgap (0.06 eV)^35^, are the promising luminescent material with long emission wavelength in NIR-IIb window. Furthermore, the high mobility of Ag ions may cause abundant cation vacancies and crystal defects^36,37^, which facilitates the element doping. Due to the self-purification of nanoparticles, the introduction of impurities into nanocrystals often requires more energy^38^. Therefore, unlike the classical QDs synthesis strategies using high temperature for nucleation and low temperature for growth^39^, the nucleation and growth of Mn/QD SAC were conducted under high temperature (180 °C) (see Methods). After the fluorescence optimization with Mn:Ag feed ratio (Supplementary Fig. 1), the Mn content in Mn/QD SAC was determined to be 0.27 ± 0.03 wt% by inductively coupled plasma emission spectrometer (ICP-AES) (Supplementary Table 1). The as-prepared Mn/QD SAC were uniform with a narrow size distribution (4.5 ± 0.4 nm) demonstrated by transmission electron microscopy (TEM) (Fig. 2b). The high-resolution TEM image indicated that the interplanar distance of 0.09 nm and 0.17 nm matched the (100) and (012) planes of monoclinic Ag_2_Te [JCPDS card, no.81–1820] respectively. The high-angle annular dark-field scanning transmission electron microscopy (HAADF-STEM) indicated that QDs were composed of Ag, Te, and Mn. The elemental mapping image clearly demonstrated that Mn atomically dispersed on Ag_2_Te QDs (Fig. 2c). Structural analysis by X-ray diffraction (XRD) further proved the composition of monoclinic Ag_2_Te, and the doping of Mn did not significantly change the shape of the Ag_2_Te QDs. Notably, no crystal-phase signals of Mn can be observed, manifesting high isolation of Mn atoms on Ag_2_Te QDs (Fig. 2d). The electron spin resonance (ESR) spectrum also displayed a symmetrical and uniform sextet (characteristic peak of Mn^2+^) (Fig. 2e). Furthermore, the hyperfine coupling constant reflecting chemical environment of Mn^2+^ was calculated to be 91 G, which could be assigned to surface Mn^40,41^. The above results confirmed that Mn^2+^ atomically distributed on the surface of nanocrystals, which were conducive to a series of antioxidant reactions.

For in vivo use, an amphiphilic polymer was coated on the surface of Mn/QD SAC to transfer them into aqueous solution according to our previous works^42,43^. Then, a cross-linked layer was composited by conjugating branched polyethylene glycol (PEG) molecules on the surface of Mn/QD SAC, which enhanced the stability and biocompatibility of Mn/QD SAC in biological media. Dynamic light scattering (DLS) measurement showed that the hydrodynamic size of Mn/QD SAC increased from 10.4 to 23.6 nm (Supplementary Fig. 2a). After PEGylation, the zeta potential changed from −20.13 to 1.50 mV (Supplementary Fig. 2b). TEM image suggested that the modified Mn/QD SAC can evenly disperse in PBS buffer with no obvious aggregation (Supplementary Fig. 3). In addition, Mn/QD SAC could be stored in PBS buffer, MES buffer, and fetal bovine serum (FBS) for 30 days without obvious aggregation (Supplementary Fig. 4). Next, the optical properties of Mn/QD SAC were investigated. The emission peak of Mn/QD SAC located in the NIR-IIb with the wavelength at ~1690 nm (Fig. 2f), the inset images in Fig. 2f clearly demonstrated the significant enhancement of fluorescence intensity after Mn doping (increased by ~80%). The absolute photoluminescence quantum yield (PLQY) of Mn/QD SAC was measured to be 4.02%, facilitating the application in NIR-IIb imaging in vivo. Next, the time-resolved photoluminescence decay curves were used to further analyze the reason of fluorescence enhancement. Evidently, Mn/QD SAC possessed the longer lifetime of 36.67 ns in comparison with lifetime of Ag_2_Te QDs (30.83 ns). Moreover, the short life proportion (R_1_) after Mn doping reduced from 9.07% to 5.63% (Fig. 2g). High photostability of Mn/QD SAC was observed in a long-time exposure test under an 808 nm laser (100 mW/cm^2^). The fluorescent intensity of Mn/QD SAC remained strong with a decrease less than 2%, which could prevent the image distortion caused by fluorescence decay. In contrast, ICG suffered a fluorescence decay by ∼90% after irradiation (Fig. 2h, i). The above results indicated that Mn^2+^ may act as electron donors and fill the surface defects of Ag_2_Te QDs, thus suppressing the nonradiative transition and improving the fluorescence performance^36,44^.

### ROS scavenging activity of Mn/QD SAC

**Fig. 3:**
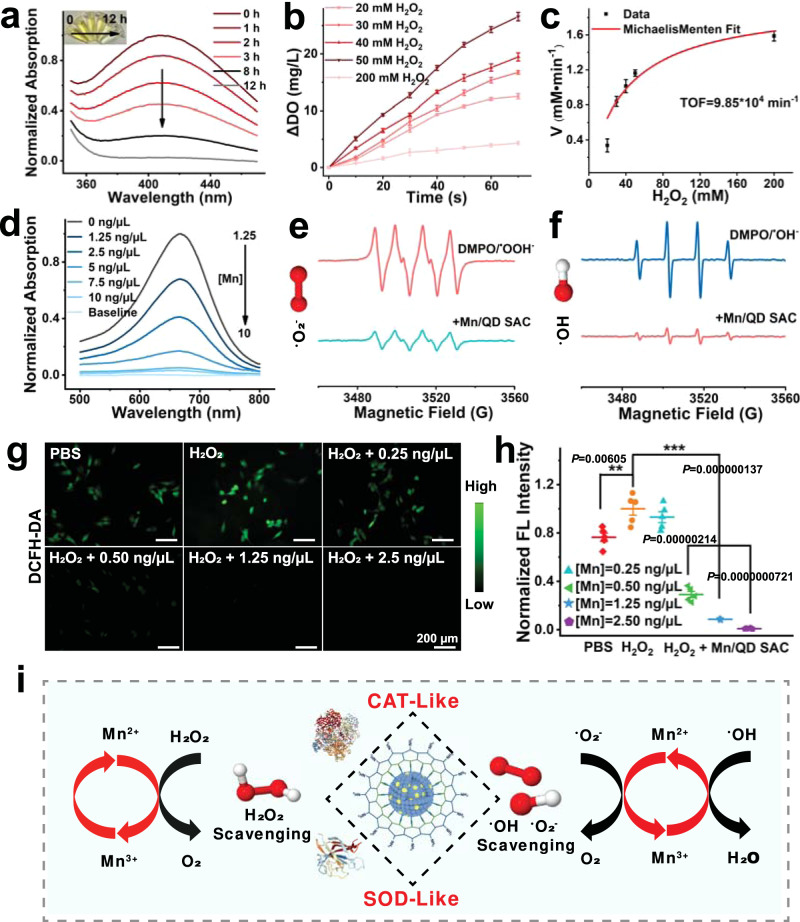
Enzyme-mimetic and ROS scavenging activity of the Mn/QD SAC. **a** Time-dependent absorption spectrum with Mn/QD SAC in titanium sulfate colorimetry method. **b** Oxygen generation in various concentration of H_2_O_2_ solutions with Mn/QD SAC. Data are presented as mean ± SD (*n* = 3 independent samples). **c** Steady-state kinetic analysis for the CAT-like activity of Mn/QD SAC. The TOF were obtained from the Michaelis–Menten equation. Data are presented as mean ± SD (*n* = 3 independent samples). **d** Concentration-dependent absorption spectrum with Mn/QD SAC for the ^•^O_2_^−^ scavenging in NBT method. **e** ESR spectra of ^•^O_2_^−^ with Mn/QD SAC using DMPO as the spin trap agent. **f** ESR spectra of ^•^OH with Mn/QD SAC using DMPO as the spin trap agent. **g** Representative fluorescence images of total ROS levels in HT22 cells induced by H_2_O_2_ in the presence of Mn/QD SAC with different concentrations. **h** Semi-quantitative analysis of relative fluorescence intensity of cells corresponding to panel **g**. Data are presented as mean ± SD (*n* = 5 independent samples). **i** Schematic illustration of CAT-like and SOD-like catalytic activity of Mn/QD SAC for various ROS scavenging. Statistical significance was calculated via two-tailed two-sample Student’s *t* test (**h**). (**) *P* < 0.01; (***) *P* < 0.001. Source data are provided as a Source Data file.

H_2_O_2_, ^•^O_2_^−^, and ^•^OH, as the main ROS in vivo, are always produced in large quantities and cause serious injury to neurons after TBI^11^. Therefore, the effective ROS elimination can significantly alleviate neuroinflammation and repress the further BBB breakdown^16^. Among them, H_2_O_2_ is of greatest importance due to the highest intracellular concentration, membrane permeability and longer half-life than ^•^O_2_^−^ and ^•^OH^19,45^. The H_2_O_2_ scavenging capacity of Mn/QD SAC was investigated with titanium sulfate spectrophotometric (TSS) and measuring the change of dissolved oxygen (DO) after H_2_O_2_ decomposition respectively. Generally, 80% to 90% H_2_O_2_ (1 mM) can be scavenged by 400 ng/µL of commonly used antioxidant vitamin C^46^, whereas Mn/QD SAC ([Mn] = 10 ng/µL) could continuously scavenge 20 mM of H_2_O_2_ overtime with an elimination rate reaching to ~98% (Fig. 3a), indicating the high CAT-like activity of Mn/QD SAC (about 1000-fold than vitamin C). The steady-state kinetic analyses showed that Mn/QD SAC obeyed the typical Michaelis–Menten kinetics^19,47^ (Fig. 3b, c). The turnover frequency (TOF) of enzymes represents the transformation number of substrates with a single active center per unit time. Notably, the TOF of Mn/QD SAC was calculated to be 9.85 × 10^4 ^min^−1^ from Michaelis–Menten equation, almost ~3.8 × 10^4^ times higher than Mn_3_O_4_ nanoparticles (2.55 min^−1^)^48^. The K_m_ and K_cat_ for H_2_O_2_ of Mn/QD SAC calculated by Michaelis–Menten equation are 40.8 mM and 1.6 × 10^3 ^s^−1^ respectively. Mn/QD SAC exhibited strong CAT-like activity may relate to its surface single-atomic dispersed Mn with high atomic utilization rate. In addition, even under extreme temperature (4–80 °C) and pH conditions (pH = 4–11), the Mn/QD SAC still maintained the catalytic activity for effective H_2_O_2_-scavenging (Supplementary Fig. 5), which was advantageous for achieving stable ROS scavenging in the complex inflammatory environment after TBI in vivo.

Next, the scavenging ability of Mn/QD SAC for ^•^O_2_^−^ and ^•^OH was also tested. Nitro-blue tetrazolium (NBT) can react with ^•^O_2_^−^ to form a blue formazan with the absorption at 680 nm^49^. As shown in Fig. 3d, the absorption at ~680 nm reduced as the concentration of Mn/QD SAC increased, indicating ^•^O_2_^−^ could be eliminated at the concentration of [Mn] = 10 ng/µL (elimination rate was up to ~97%), the half effective concentration (EC_50_) for ^•^O_2_^−^ was 0.43 ng/µL (Supplementary Fig. 6), almost 23-fold SOD activity of CeO_2_ (10 ng/µL)^50^, showing the effective ^•^O_2_^−^ remove effect and high SOD-like activity of Mn/QD SAC. In addition, the ability to scavenge ^•^O_2_^−^ was further verified by electron spin resonance (ESR). DMPO, as a free radical trap agent, can combined with ^•^O_2_^−^ to form the DMPO/^•^OOH^−^^20^. After adding Mn/QD SAC, the signal of DMPO/^•^OOH^−^ reduced by 72%, indicating the effective elimination of ^•^O_2_^−^ (Fig. 3e). Similarly, DMPO can react with ^•^OH to produce the DMPO/^•^OH^20^, as shown in Fig. 3f, the intensity of DMPO/^•^OH decreased by 81% after incubation with Mn/QD SAC, indicating the scavenging of ^•^OH.

The cellular anti-ROS effect of the Mn/QD SAC was also evaluated with mouse hippocampal neuron cells (HT22) in vitro. Imaged by ROS probes (DCFH-DA), the intracellular ROS level rose up evidently after incubating with H_2_O_2_, but gradually decreased after the addition of Mn/QD SAC (Fig. 3g). In the presence of 2.50 ng/µL of [Mn], nearly 99% of intracellular ROS could be eliminated (Fig. 3h). The effective ROS scavenging and enzyme-like activities may originate from the highly dispersed single-atom Mn near the surface layer of Ag_2_Te nanocrystals to increase the contact possibility with ROS (Fig. 3i).

### Monitoring of BBB Breakdown after TBI by NIR-IIb Imaging

**Fig. 4:**
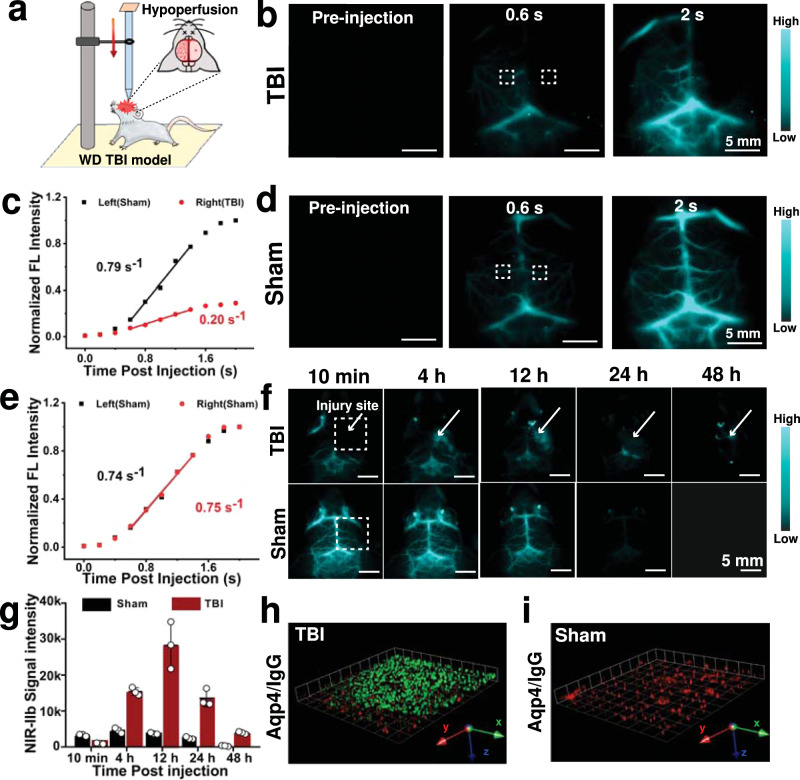
Dynamic and high-resolution NIR-IIb imaging for monitoring the status of BBB in acuate phase of TBI. **a** Schematic displaying the experimental procedure for the WD TBI model. **b** Dynamic NIR-IIb image frames recorded at indicated time points post injection of Mn/QD SAC with TBI mouse. The ROI is outlined by white dotted lines. **c** NIR-IIb signals in the left (contralateral) and right (ipsilateral) cerebral hemispheres of the TBI mouse versus time according to the panel **b**. **d** Dynamic NIR-IIb image frames recorded at indicated time points post injection of Mn/QD SAC with healthy mouse with sham treatment. The ROI is outlined by white dotted lines. **e** NIR-IIb signals in the left (contralateral) and right (ipsilateral) cerebral hemispheres of the healthy mouse versus time according to the panel **d**. **f** Dynamic monitoring of TBI mouse brain with NIR-IIb imaging. **g** Time course of NIR-IIb signals in sham and TBI mouse in the healthy and injured ROI. The ROI is outlined by white dotted lines in panel **f**. Data are presented as mean ± SD (*n* = 3 independent samples). 3D reconstruction of the fluorescent images obtained in serial optical sections showing IgG (Green) extravasation in the mice with TBI in panel **h** but not in the sham control in panel **i**. Source data are provided as a Source Data file.

The TBI was induced in the right side of the head of wild type BALB/c mice (age 8 weeks) with a weight-drop (WD) method (Fig. 4a)^51^. Three hours post-TBI (p.t.), Mn/QD SAC were intravenously injected for in vivo imaging (exposure time: 150 ms) of brain without craniotomy (Fig. 4b). The NIR-IIb signals of the cerebral transverse sinus, middle cerebral vessels, inferior cerebral vein in left brain showed up immediately within 0.6 s post-injection (p.i.), and rapidly increased and plateaued in 2 s p.i., whereas, the signals on the right side (injury side) were nearly unnoticeable, displaying the wide “black area” (Supplementary Movie 1 and Fig. 4b). The fluorescence intensity of region of interest (ROI) in the right brain at 2 s p.i. was only 28.9% of that in the corresponding left side. Analysis for dynamic fluorescence signals showed that cerebral blood perfusion rate of injury side (0.20 s^−1^) was roughly 1/4 of that in corresponding left side (0.79 s^−1^) of ROI (Fig. 4c). Conversely, in healthy mice with sham injury, the NIR-IIb signals of both cerebral hemispheres showed up simultaneously (Supplementary Movie 2 and Fig. 4d) and displayed identical perfusion rates (0.74 s^−1^ and 0.75 s^−1^ respectively) (Fig. 4e). The above results indicated that a considerable reduction in cerebral blood flow occurred after TBI, which may lead to ischemic injuries and neurological dysfunction^52^. Notably, a small vessel with an apparent width of ~9.6 μm could be unambiguously observed in NIR-IIb window (Supplementary Fig. 7a, b), confirming the high resolution for cerebral endothelial capillaries (7–12 μm). The high spatiotemporal resolution of NIR-IIb imaging facilitated the investigation of cerebral hemodynamics after BBB breakdown and further understanding the mechanism of rapid response to damage.

Cerebral microvascular injury is usually in dynamic changes during the acute phase of TBI^53^, which should be closely monitored to detect clinical deterioration in time. Owing to the long circulation time of Mn/QD SAC in blood (Supplementary Fig. 8a–c), we continuously monitored the status of BBB in 48 h p.i. As shown in Fig. 4f, NIR-IIb signals in sham injury mouse brain decreased over time and successfully eliminated from the blood circulation within 48 h without any leakage through the intact BBB. In contrast, NIR-IIb signals in the injury side brain of TBI mice enhanced cumulatively and exhibited a diffusion trend with the strongest signal at 12 h p.i. (Fig. 4g). The obvious accumulation signals were still observed even at 48 h p.i., indicating the serious leakage through impaired BBB and further trap into brain tissues^52^. The signal background ratio (SBR) of the leakage site reached up to 14.3 (far beyond the Rose criterion SBR > 5) (Supplementary Fig. 9a, b)^54^, indicating a high sensitivity for the detection of cerebrovascular injury lesions after TBI in a noninvasive way.

Ex vivo imaging of the whole brain from sacrificed TBI mice also showed strong NIR-IIb signals at the injury site (Supplementary Fig. 10a). The immunostaining of brain tissue slices confirmed the microvascular injuries with the up-regulated expression of aquaporin-4 (Aqp4) (Supplementary Fig. 10b). In addition, the continuous optical scanning and three-dimensional reconstruction images of brain slices displayed that a large amount of plasma IgG (a standard marker of BBB destruction)^52^ leaked into brain tissues at 24 h p.t. (Fig. 4h), but not in the sham control mouse (Fig. 4i). The above results were highly consistent with our in vivo noninvasive NIR-IIb imaging data of cerebral microvascular injuries to demonstrate the BBB impairment after TBI.

### Neuroprotective and anti-inflammation effects of Mn/QD SAC

**Fig. 5:**
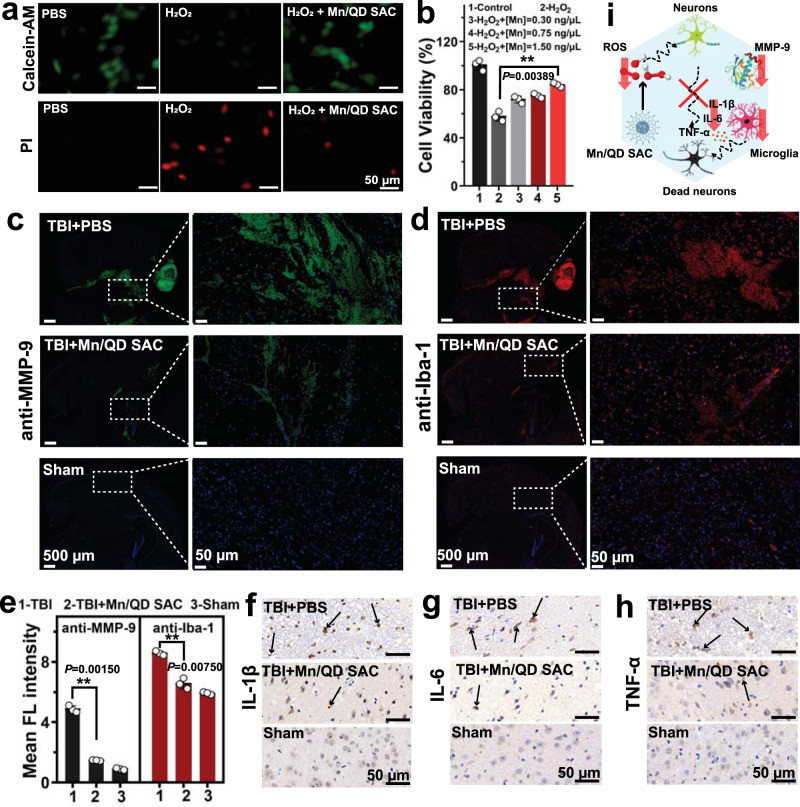
Neuroprotective and anti-inflammation effects of the Mn/QD SAC for TBI mice. **a** Representative fluorescence images of calcein-AM (Green, live cells) and propidium iodide (Red, dead cells) co-stained HT22 cells after different treatment. **b** Cell viability of HT22 cells treated with H_2_O_2_ and different concentrations of Mn/QD SAC measured by CCK-8 method. Data are presented as mean ± SD (*n* = 3 independent samples). **c** Representative immunostaining images of the brain sections TBI with anti-MMP-9 (Green) for matrix metalloproteinase-9. The immunostaining images were obtained from the digital slide scanner equipped with 10× objective. **d** Representative immunostaining images of the brain sections with anti-Iba-1 (Red) for microglia. The immunostaining images were obtained from the digital slide scanner equipped with 10× objective. **e** Semi-quantitative analysis of mean fluorescence intensity of mouse brain slices corresponding to panel **c** and panel **d**. Representative immunohistochemistry images for IL-1β (**f**), IL-6 (**g**), and TNF-α (**h**) in the mouse brain slices at 12 h post TBI. **i** Schematic illustration of Mn/QD SAC for reducing the expression of MMP-9, inhibiting microglia activation and the release of pro-inflammatory cytokine to protect nerve cells. Data are presented as mean ± SD (*n* = 3 independent samples). Statistical significance was calculated via two-tailed two-sample Student’s *t* test (**b** and **e**). (**) *P* < 0.01. Source data are provided as a Source Data file.

The long-term and excessive inflammatory reaction, as the important process of secondary injury after TBI, often lead to poor clinical outcomes caused by neurons loss and death^5,13^. The neuroprotective and anti-inflammation effect of Mn/QD SAC were investigated in vitro. After incubating HT22 cells with various concentrations of Mn/QD SAC in the culture medium containing H_2_O_2_ for 24 h, the in vitro cell staining with calcein-AM (green, live cells) and PI (red, dead cells) showed that Mn/QD SAC greatly relieved the ROS-induced death of HT22 cells (Fig. 5a). This result was also verified by cell viability tests with the Cell-Counting-Kit-8 (CCK-8) method (Fig. 5b). In the presence of Mn/QD SAC ([Mn] = 1.5 ng/µL), the cell viability remained over 85%, while that of the control group decreased to 58%, indicating the good neuroprotective effect of Mn/QD SAC.

Subsequently, the in vivo anti-inflammatory activity and neuroprotective effect of Mn/QD SAC were studied with TBI mouse models. The MMP-9 and activated microglia are commonly used as biomarkers to identify brain inflammation^55–57^. The immunostaining of brain slices from TBI mice with Mn/QD SAC treatment showed that the expression of MMP-9 and Iba-1(marker of activated microglia) decreased by 70% and 24%, respectively (Fig. 5c–e). In addition, three key pro-inflammatory factors (IL-1β, IL-6, and TNF-α) released by microglia were also significantly reduced as shown in the immunohistochemistry images (Fig. 5f–h). This result was also verified by enzyme-linked immunosorbent assay (ELISA) that the three factors reduced by 63%, 31%, and 55% respectively after treatment with Mn/QD SAC (Supplementary Fig. 11), indicating that inflammatory responses were evidently relieved after the scavenging of ROS. In addition, the apoptosis staining of brain tissues was conducted to study the neuroprotective effect of Mn/QD SAC in vivo 24 h p.t., widespread apoptotic cells was observed near the injury site of brain. However, Mn/QDs SAC can significantly reduce the signals of apoptosis (Supplementary Fig. 12), indicating the neuroprotective effect for TBI treatment. Notably, we found that less brain edemas occurred in TBI mice with Mn/QD SAC treatment (Supplementary Fig. 13). The above results indicated that Mn/QD SAC markedly reduced the expression of MMP-9 and inhibited the release of pro-inflammatory cytokines by scavenging various ROS (Fig. 5i), thus alleviating ROS-mediated neuroinflammation and brain edemas for BBB reconstruction.

### NIR-IIb imaging-guided therapy to promote the recovery of BBB after TBI

**Fig. 6:**
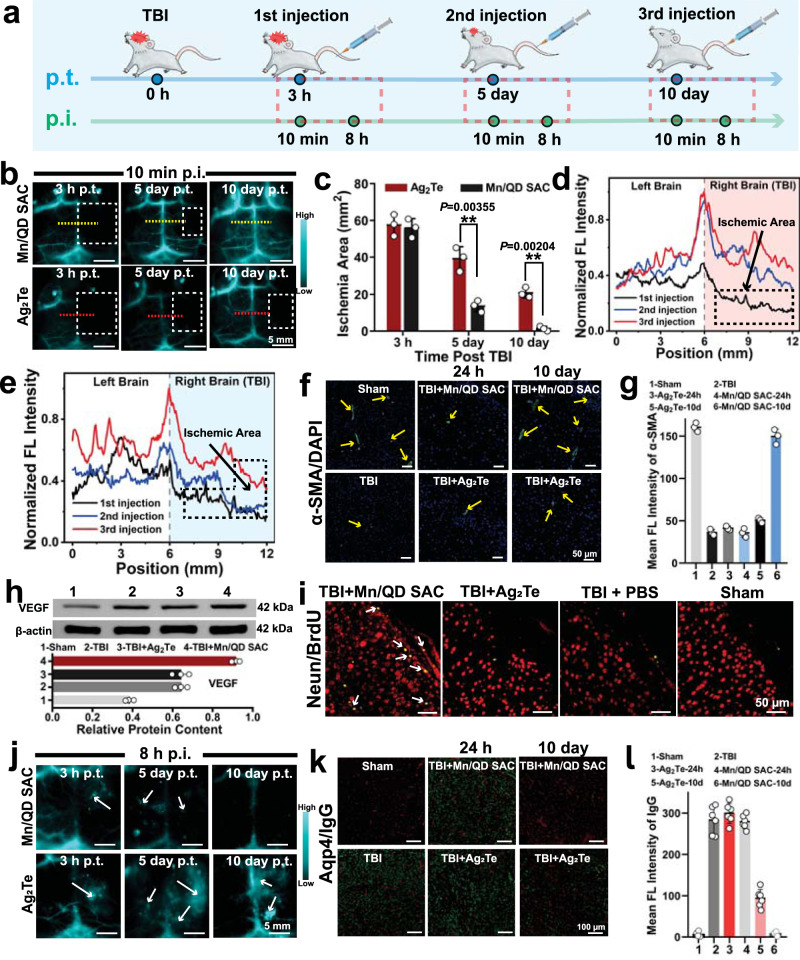
NIR-IIb imaging-guided ROS scavenging therapy by monitoring BBB status and promoting BBB recovery with Mn/QD SAC for TBI. **a** Schematic showing the different treatments on BALB/c mice. **b** NIR-IIb imaging of the brain of TBI mice performed within 10 min post injection with Mn/QD SAC or Ag_2_Te at 3 h, 5-th, or 10-th day post TBI. The white dotted line frames the ischemic areas of the brain. **c** Size changes of brain ischemic regions over time in TBI mice with different treatments. Data are presented as mean ± SD (*n* = 3 independent samples). **d** NIR-IIb intensity profiles of the brain along yellow dashed lines in panel **b** of Mn/QD SAC group. **e** NIR-IIb intensity profiles of the brain along red dashed lines in panel **b** of Ag_2_Te group. **f** Representative immunostaining images of the brain sections with anti-α-SMA for α-SMA (Green). The yellow arrow points to the location of brain micro-vessels. **g** Corresponding semi-quantitative fluorescence analysis of α-SMA expression in panel **f**. Data are presented as mean ± SD (*n* = 3 independent samples). **h** Western blot analysis of VEGF expression in mice brains with different treatments. Data are presented as mean ± SD (*n* = 3 independent samples). **i** Representative immunostaining images in mice brains with anti-BrdU/anti-Neun co-staining for newborn neurons. The white arrows point to the location where BrdU (Yellow, the markers of cell proliferation) and Neun (Red, the markers of neurons) are co-expressed. **j** NIR-IIb imaging of the brain of TBI mice performed within 8 h post injection with Mn/QD SAC or Ag_2_Te at 3 h, 5-th, or 10-th day post TBI. The white arrow points to the location of leakage. **k** Representative immunostaining images of the brain sections with anti-IgG for mouse plasma IgG (Green) and anti-Aqp4 for aquaporin-4 (Red). **l** Corresponding semi-quantitative fluorescence analysis of plasma IgG expression in panel **k**. Data are presented as mean ± SD (*n* = 6 independent samples). Statistical significance was calculated via two-tailed two-sample Student’s *t* test (**c**). (**) *P* < 0.01. Source data are provided as a Source Data file.

The mice were injected with Mn/QD SAC at different time points (3 h, 5 days, and 10 days p.t.) to evaluate the recovery of BBB (Fig. 6a). The PEGylated Ag_2_Te QDs was also used as control without ROS-scavenging ability. After first injection (3 h p.t.), TBI mice treated with Mn/QD SAC or Ag_2_Te both showed the similar size of ischemic areas in the right brains (Fig. 6b). However, on the 5-th day p.t. (with second injection), the ischemia areas in Mn/QD SAC-treated mice significantly reduced by 76% (from 55 to 13 mm^2^), and almost no hypoperfusion areas left on the 10-th day p.t. (with third injection) (Fig. 6c). The fluorescence intensity analysis of the left and right brains confirmed the good recovery of blood perfusion on the injured side after Mn/QD SAC treatment (Fig. 6d). In contrast, large hypoperfusion areas still existed in the right brain of Ag_2_Te-treated mice even on the 10-th day p.t. (Fig. 6b, e). Due to the effective scavenging of ROS by Mn/QD SAC, the TBI mice could quickly recover the normal brain blood perfusion. Furthermore, the precise spatiotemporal assessment of cerebral ischemia provided by NIR-IIb imaging can guide appropriate treatment options to prevent long-term ischemia injuries.

We also analyzed the molecular expression related to the recovery of vessels in the brain during the treatment. Owing to cerebral vessels injuries caused by external forces, the expression of alpha-smooth muscle actin (α-SMA, typically expresses in vascular smooth muscle cells) in the brain dropped dramatically (Fig. 6f)^58^. However, after the treatment with Mn/QD SAC, the expression of α-SMA in the brain up-regulated evidently, resulting in higher cerebrovascular density than the control group on the 10-th day p.t. (Fig. 6f, g). Further, the western blot analysis indicated that the expression of vascular endothelial growth factor (VEGF) dramatically up-regulated in TBI mice treated with Mn/QD SAC (Fig. 6h), indicating the formation of new vessels^59,60^. Moreover, the co-staining of BrdU (the marker of cell proliferation) and Neun (specific nucleoproteins of vertebrate neurons) in newborn neuronal cells showed that TBI mice treated with Mn/QD SAC displayed a higher level of nerve regeneration (Fig. 6i)^61^. The angiogenesis and neurogenesis processes boosted by Mn/QD SAC contributed to the rapid recovery of BBB functions.

We further studied the reconstruction of tight junction at BBB by long-time NIR-IIb imaging. As shown in Fig. 6j, wider range of accumulation of NIR-IIb signals occurred in the right brain of mice injected with Ag_2_Te at the 5-th day p.t., and the persistent leakage still did not disappear even at the 10 th day p.t. In contrast, there were only a few fluorescent spots in the brain of mice treated with Mn/QD SAC on the 5-th day p.t., the leakage phenomenon completely terminated on the 10-th day p.t. (Fig. 6j), which was consistent with the immunofluorescence staining results and the corresponding semi-quantitative analysis in the brain tissues (Fig. 6k, l). Moreover, the expression of zonula occludens 1 (ZO-1, reflects the degree of tight junction between cells) ^62^ also confirmed the recovery of BBB integrity (Supplementary Fig. 14a, b). Through the above results, due to the satisfactory NIR-IIb imaging performance and anti-inflammation effect of Mn/QD SAC, the BBB recovery status could be monitored dynamically with high-resolution in a non-invasive way, the timely curative effect feedback can be obtained to achieve the more accurate and effective treatment for TBI.

### Animal behavior test and biosafety evaluation

**Fig. 7:**
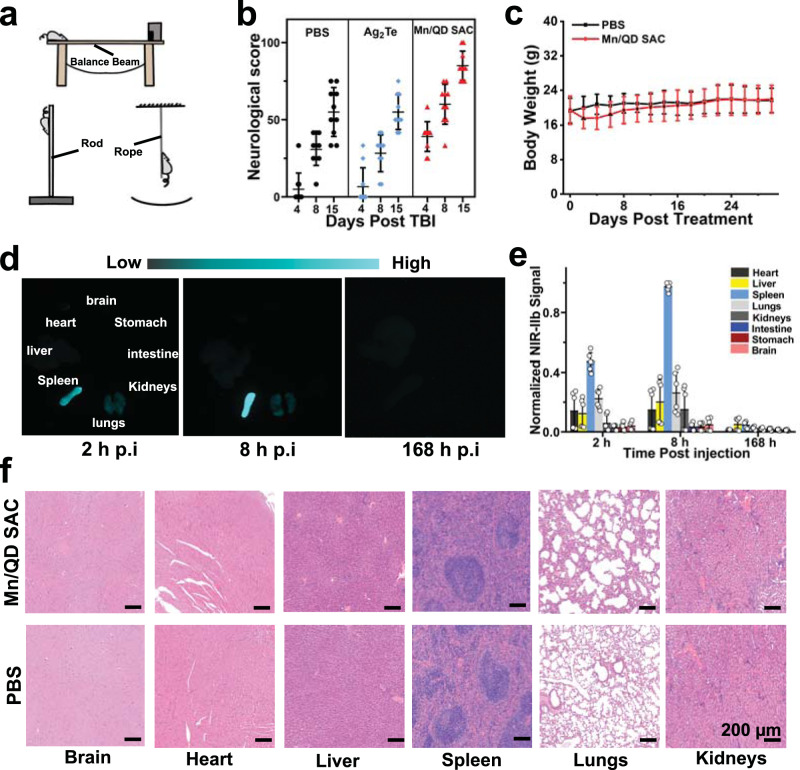
Neurological score and biosafety investigation. **a** Schematic diagram of balance beam experiment, climbing rod experiment and suspension rope experiment. **b** Comprehensive neurological score based on exercise ability tests. Data are presented as mean ± SD (*n* = 10 independent samples). **c** Body weight of mice after different treatments within 30 days. Data are presented as mean ± SD (*n* = 6 independent samples). **d** In vitro NIR-IIb fluorescence imaging of the main tissues of mice harvested at different time points (2, 8, and 168 h) post injection of Mn/QD SAC. **e** Semi-quantitative analysis of normalized NIR-IIb fluorescence intensity for tissue distribution of Mn/QD SAC at different time points post injection. Data are presented as mean ± SD (*n* = 6 independent samples). **f** Representative H&E staining images of the organs harvested from mice at 30 days post injection of Mn/QD SAC. Source data are provided as a Source Data file.

To further investigate the neuroprotective effect of Mn/QD SAC, three conventional animal behavioral tests (the balance beam, climbing pole and suspension rope experiment) were conducted on TBI mice (Fig. 7a)^63,64^. One-week training for mice was performed before TBI to ensure the validity of tests. The mice in each group gradually mastered the test items and demonstrated the similar exercise ability (Supplementary Fig. 15a–c). The rules were formulated for neurological scoring based on above tests (Supplementary Tables 2–4). After different treatments, behavioral tests were conducted on mice at the 4-th, 8-th, and 15-th day p.t. At the 4-th day p.t., all groups of mice showed a significant decline in motor ability (Supplementary Fig. 15d–f). However, the TBI mice with Mn/QD SAC treatment recovered more than 60% of the exercise capacity only after 8 days (Supplementary Movie 3–5). After 15 days, TBI mice treated with Mn/QD SAC obtained the highest scores compared with other groups, and recovered ~ 85% of the exercise capacity (Fig. 7b).

To prove the possibility of clinical translation, the biocompatibility of Mn/QD SAC was systematically investigated. The cytotoxicity of Mn/QD SAC to HT22 cells and mouse brain microvascular endothelial cells (bEnd.3) was evaluated by the CCK-8 method. After incubating cells with different concentrations (0, 25, 50, 100, 250, and 500 μg/mL) of Mn/QD SAC for 24 h, the absorbance at 450 nm was recorded by the enzyme microplate reader to evaluate the cell viability. Two cell lines still maintained good cell viability when the concentration of Mn/QD SAC was 500 μg/mL (Supplementary Fig. 16), indicating that Mn/QD SAC possess no obvious cytotoxicity to living cells. The hemolytic test was conducted to study the in vivo biosafety of Mn/QD SAC. At the concentration of 0.4 mg/mL (similar to that in the blood in vivo when used for imaging-guided therapies), the Mn/QD SAC did not cause obvious hemolysis even after 24 h (Supplementary Fig. 17). Further, biochemical analysis of blood and hematoxylin and eosin (H&E) staining of major organs were performed to investigate the acute toxicity of the Mn/QD SAC in vivo. After the injection of the Mn/QD SAC (200 µL, 3.72 mg/mL) for 24 h, the serum biochemical indicators of mice such as alanine aminotransferase (ALT), aspartate aminotransferase (AST), alkaline phosphatase (ALP), urea nitrogen (BUN) and creatinine (CREA) were in the normal range of values^65^ (Supplementary Table 5), indicating Mn/QD SAC did not cause obvious acute injury to hepatorenal function in vivo. The results of H&E staining for main organs also did not display obvious acute injury (Supplementary Fig. 18), indicating the low toxicity of Mn/QD SAC in vivo. In addition, the body weight of healthy mice injected with Mn/QD SAC decreased slightly at first but finally reached a similar levelto that of control group, indicating the low toxicity of Mn/QD SAC in vivo (Fig. 7c). Next, the bio-distribution of Mn/QD SAC in vivo was also investigated as shown in Fig. 7d. The fluorescence semi-quantitative analysis indicated that Mn/QD SAC were mainly distributed in spleen, lungs, and liver within 8 h post injection, and almost completely excluded from the body within 7 days with a low cumulative risk in vivo (Fig. 7e). Besides, the potential toxicity to major organs after a long period of time (30 days) post injection of Mn/QD SAC was also evaluated by H&E staining, no obvious cell necrosis and inflammatory can be seen in the organs obtained from the Mn/QD SAC treated mice (Fig. 7f). Therefore, the Mn/QD SAC displayed the low toxicity and good biocompatibility in vivo.

## Discussion

At present, the treatment for patients with severe TBI mainly concentrate on the acute phase. Although relevant clinical interventions and nursing methods have been greatly improved, it is still a great challenge to reduce the secondary injury and avoid long-term neurological abnormalities after TBI. In recent years, the ROS-scavenging therapy can alleviate nerve oxidative damage after TBI and significantly improve the neurological function in animal models, showing the great potential for long-term prognostic management after TBI. However, the secondary injury events after TBI closely related to the further BBB breakdown and were usually in dynamic changes. Therefore, it is necessary to dynamically monitor the BBB status, which can be the valuable guidance for determining the injury degree and adjusting the treatment strategies in time.

In summary, we have developed a QD-based single-atom catalyst with NIR-IIb emitting ability for imaging-guided therapy of TBI. The isolated single Mn atoms on the surface of QDs exhibited high ROS-scavenging activity to alleviate neuroinflammation, down-regulate the expression of MMP-9, and inhibit the release of pro-inflammatory cytokines after TBI. By dynamically monitoring the variations of BBB status through noninvasive NIR-IIb imaging with high spatiotemporal resolution, we found that continuous BBB breakdown caused by secondary injury was significantly recovered after the scavenging of ROS, which was verified by the upregulated molecular expression of ZO-1, α-SMA, and VEGF. Overall, rapid reconstruction of BBB and recovery of neurological function were realized in 10 days post TBI under the guidance of real-time NIR-IIb imaging without craniotomy. At present, nanoparticle-based ROS scavengers with antioxidant activity have been developed to alleviate inflammation and reduce ROS-mediated oxidative damage in vivo, showing good prospects for the therapy of TBI and other brain diseases. However, lack of targeting ability limits the delivery efficiency of the nano agents into brains. In the future, by further improving the BBB-crossing and specific targeting abilities of our current single-atom catalyst, this NIR-IIb imaging-guided therapy would provide a feasible solution for the treatment of ROS-mediated brain diseases.

## Methods

### Ethics statement

Ethical approval of this study was obtained from the Animal Ethics Committee of the School and Hospital of Stomatology, Wuhan University (approval number: S07920070E). All animal experimental procedures were performed in accordance with the Regulations for the Administration of Affairs Concerning Experimental Animals approved by the State Council of the People’s Republic of China.

### Materials

Silver acetate (AgAc, 99%, powder) was purchased from Alfa. Tellurium (Te, 99.9%, powder) was purchased from Aladdin. Manganese (II) acetate tetrahydrate (Mn(Ac)_2_, 99.99%, powder), Trioctylphosphine (TOP, 97%), 1-octylmercaptan (98.5%), N,N’-dicyclohexylcarbodiimide (DCC), poly(acrylic acid) (PAA, MW~1800, 99%), N-(3-dimethylaminopropyl)-N′-ethylcarbodiimide hydrochloride (EDC) were purchased from Sigma–Aldrich. 1-octadecene (ODE, 90%) was purchased from Alfa. Trichloromethane (99%), tetrachloroethylene (TCE, 99.5%), absolute ethanol (99.8%), methanol (99.5%), acetone (99.5%), anhydrous sodium carbonate (Na_2_CO_3_, 99.8%), hydrogen peroxide (H_2_O_2_, 30%) were purchased from China National Pharmaceutical Group Corporation. Titanium Sulfate (Ti(SO_4_)_2_, 99.5%), ferrous sulfate (FeSO_4_, 99.5%), Xanthine (X, 98%) and 5,5-Dimethyl-1-pyrroline-N-oxide (DMPO) were purchased from Meryer Chemical Technology Co., Ltd. Xanthine oxidase (XO) was purchased from ShanghaiyuanyeBio-TechnologyCo,Ltd. PEG-amine (MW~5k) and 8-Arm PEG-amine (MW~40 K) were purchased from Advanced BioChemicals.

### Synthesis of Mn/QD SAC and Ag_2_Te QDs

Mn/QD SAC are composed of Ag precursor, Mn dopant, and Te precursor. Ag_2_Te are composed of Ag precursor and Te precursor. Te precursor solution was prepared by mixing 50 mg (0.39 mmol) of Te powder and 3 mL of TOP in two-neck flask at 150 °C under argon for 1 h. Ag precursor solution was prepared by mixing 65 mg (0.39 mmol) of AgAC, 10 mg of Mn(Ac)_2_ (0.06 mmol) and 8 mL of oleylamine in a three-neck flask and degassing for 1 h under argon at room temperature and then increased to 180 °C rapidly, 1 mL Te precursor was quickly injected into the three-necked flask, then reacted for 5 min, finally terminated the reaction with cold ethanol, then dissolved the participate with n-hexane after centrifugation.

### Modification of Mn/QD SAC and Ag_2_Te QDs with oleyamine-branched polyacrylic acid (OPA)

Firstly, OPA was synthesized according to our previous work^42^. Typically, 2.7 g of poly(acrylic acid) powder (average MW~1800) and DCC (4.68 g) were placed into a round-bottom flask. 30 mL of DMF was added to dissolve the mixture. 3.6 mL of oleylamine were added dropwise into the reaction flask and then stirred overnight. The molar ratio of oleylamine to PAA is 30%. After that, 50 mL HCl (0.5 M) were added to the reaction solution. The precipitate was separated by centrifugation and re-dissolved in 3 mL methanol solution. And then 20 mL 1 M HCl was added to the solution. The precipitate was separated by centrifugation again. This procedure was repeated at least 5 times. After several purifications, the final precipitate was dissolved in 5 mL chloroform and washed by 10 mL HCl (1 M). The organic phase was collected and dried over by anhydrous Na_2_SO_4_. Finally, the chloroform was removed under vacuum, and the white solid was collected.

For surface modification, as-synthesized Mn/QD SAC or Ag_2_Te QDs (5.0 mg) were dissolved in 2.0 mL chloroform containing 20 mg of OPA. The mixture was sonicated for 5 min and the solvent was removed under vacuum by a rotary evaporator. The residue was then dissolved in 2 mL of 50 mM sodium carbonate solution under the sonication. The mixtures were precipitated with ultracentrifuge at 416,000 *g* for 1 h, and washed three times with deionized water. The purified product was dissolved in pH 8.5 MES buffer (0.01 M) and stored at 4 °C.

### PEGylation of OPA modified Mn/QD SAC

The PEGylation of OPA modified Mn/QD SAC was performed according to our previous work^42^. The previously prepared OPA modified Mn/QD SAC or Ag_2_Te QDs (2 mg) were dissolved in 200 μL MES buffer (0.01 M, pH = 8.5). 4 mg of 8-arm PEG-amine (MW ~5 K) and 2 mg of 8-arm PEG-amine (MW ~40 K) were both dissolved in 200 μL MES buffer and gradually added to Mn/QD SAC or Ag_2_Te QDs solution by stirring. 3 mg of EDC was then dissolved in 50 μL MES buffer, shaking and dropping into Mn/QD SAC or Ag_2_Te QDs solution. The mixture reacted overnight at room temperature. The product was purified by ultra-centrifugation and finally dissolved in 1 × PBS buffer.

### Characterizations

Transmission electron microscopy (TEM) and images were obtained on a JEM-F200 electron microscope (JEOL) with an acceleration voltage of 200 kV. The crystal structure was characterized by X-ray powder diffraction (XRD, Bruker D8 Advanced X-ray diffractometer). The NIR fluorescence spectra at the range of 1200–2050 nm were measured by a Fluorolog-3 spectrofluorometer (Horiba) with an InGaAs detector (liquid nitrogen cooled) using FluorEssence v. 3.8 software, and the curves at the range of 2050–2600 nm was supplemented by gaussian fitting. UV–vis−NI absorption spectra were recorded on a spectrophotometer (UV-3600 plus, Shimadzu) with UVProbe v.2.33 software. The Zeta potentials and dynamic light scattering (DLS) data were recorded on a Malvern Nano-ZS ZEN3600 zetasizer.

### Fluorescence lifetime

Time-resolved photoluminescence decay curves were measured by acquired by a time correlated single-photon counting setup, samples were excited by a 980 nm Delta Diode light source (DD-980L, Horiba). The decay curves were fitted by the following equation: ${{{{{\rm{I}}}}}}={A}_{1} \exp (-{{{{{\rm{\tau }}}}}}/{\tau }_{1})+{A}_{2}\exp (-{{{{{\rm{\tau }}}}}}/{\tau }_{2})+{{{{{\rm{I}}}}}}_{0}$, the A_1_ and A_2_ represent the former factors, the τ_1_ and τ_2_ respectively represent the short lifetime and long lifetime. The average lifetime was calculated by the following equation: ${\tau }_{{{{{{\rm{average}}}}}}}={\sum {{{{{\rm{A}}}}}}}_{i}{\tau }_{i}^{2}/{\sum {{{{{\rm{A}}}}}}}_{i}{\tau }_{i}$, (*i* = 1, 2)^37^.

### Investigation of fluorescence stability

500 µL Mn/QD SAC (OD_808nm_ = 0.5) and ICG (OD_808nm_ = 0.5) were placed in the EP tubes respectively and irradiated with 808 nm laser at the power density of 100 mW/cm^2^ for 2 h^42^. During the irradiation, the Mn/QD SAC and ICG were imaged by A 10× Mitutoyo Plan Apo NIR Infinity Corrected Objective (Edmund Optics) on NIR-II setup every 30 min. The fluorescence intensity changes were analyzed according to the FL images.

### CAT-like activity of Mn/QD SAC

The CAT-like catalytic activity of Mn/QD SAC was investigated via two methods.

Dissolved Oxygen (DO) measurement: The decomposition of H_2_O_2_ will produce O_2_. The DO in the solution was monitored to reflect the removal of H_2_O_2_^66^. The higher DO rose, the stronger CAT-like activity of Mn/QD SAC was. DO was measured by a portable dissolved oxygen meter. Add Mn/QD SAC ([Mn] = 0.25 ng/µL) to the 10 mL beaker, insert the probe of the dissolved oxygen meter below the tested liquid level, waiting for the indicator to stabilize for 5 min with the magnetic stirring, then add different concentrations of H_2_O_2_ (20, 30, 40, 50, and 200 mM) into the solution and observe the changes of DO over time.

Titanium sulfate spectrophotometric (TSS): TTS is a classic colorimetry method for detecting H_2_O_2_. Titanium sulfate will react with H_2_O_2_ to form a titanium-peroxide composite precipitate, which can be dissolved in a strong acid solution with a characteristic absorption peak at ~410 nm. The concentration of the product can be directly judged by the color. And there is a linear relationship with the H_2_O_2_ in a certain concentration range, the absorbance can be measured by an UV–vis spectrophotometer^67^. Firstly, prepare H_2_O_2_ detection working solution according to the following materials: 1.25 g Ti(SO_4_)_2_, 8 mL H_2_SO_4_ and 100 mL deionized water. Then add Mn/QD SAC ([Mn] = 10.0 ng/µL) to 20 mM H_2_O_2_ under magnetic stirring. 20 µL reaction solution was added into 1 mL previously prepared titanium sulfate solution at different time points (1, 2, 3, 8, and 12 h), the mixture was centrifuged for 20 min (6740 *g*) and the supernatant was taken to measure the UV–Vis absorption spectrum with a scanning range of 340–480 nm.

### SOD-like activity of Mn/QD SAC

The SOD-like catalytic activity of Mn/QD SAC was investigated via two methods. The both methods detected the scavenging of superoxide radicals (^•^O_2_^−^). The more ^•^O_2_^−^ were scavenged, the higher SOD-like activity of Mn/QD SAC was. In both methods, the following steps were used to produce ^•^O_2_^–^: Xanthine (X) and xanthine oxidase (XO) were mixed and incubated at 37 °C for 10 min.

Electron spin resonance (ESR): ESR was used to detect the scavenging of ^•^O_2_^−^^67^. Firstly, the Mn/QD SAC (12.5 ng/µL) were mixed with X (5 µM) and XO (0.5 U/mL) to react for 10 min at 37 °C, then 10 µL DMPO was added into the mixture to trap the remaining ^•^O_2_^−^, and the ESR spectrum was quickly measured under the room temperature. The DMPO/^•^OOH^−^ yielded four characteristic lines with relative intensities of 1:1:1:1.

Nitro-blue tetrazolium colorimetry (NBT) method: NBT colorimetry is a classical method for the detection of ^•^O_2_^−^. The darker the color of the reaction solution, the more ^•^O_2_^−^ exists in the system. The absorbance can be measured by an UV–vis spectrophotometer. Firstly, various concentrations of Mn/QD SAC (1.25, 2.5, 5, 7.5, and 10 ng/µL) were mixed with X (40 mM), NBT (1 mM) and XO (1 U/mL) to react for 20 min at 37 °C. The mixture was centrifuged for 20 min (6740 *g*), discarded the supernatant, then added 1 mL 1 × PBS to washed the precipitation and centrifuged for another 20 min (6740 *g*), repeated the washing process for at least twice. Finally, the precipitate was dissolved in DMSO (1 mL) and the solution was taken to measure the UV–Vis absorption spectrum with a scanning range of 500–800 nm.

### Hydroxyl radical scavenging activity of Mn/QD SAC

Fenton reactions are used to produce ^•^OH. Electron spin resonance (ESR) was used to detect the scavenging of ^•^OH^67^. Firstly, the Mn/QD SAC ([Mn] = 12.5 ng/µL) were mixed with FeSO_4_ (20 µM) and H_2_O_2_ (10 mM) to react for 2 min, then 10 µL DMPO was added into the mixture to trap the remaining ^•^OH, and the ESR spectrum was quickly measured under the room temperature. The DMPO/^•^OH yielded four characteristic lines with relative intensities of 1:2:2:1.

### Investigation of catalytic stability

Temperature conditions:1 mL Mn/QD SAC ([Mn] = 10 ng/µL) and CAT (1 U/mL) were placed in 4, 20, 50, and 80 °C environment respectively for 48 h. 20 mM H_2_O_2_ was added to reaction for 3 h. Titanium sulfate colorimetry was used to detect residual H_2_O_2_. 20 µL reaction solution was added into 1 mL titanium sulfate solution, the mixture was centrifuged for 20 min (6740 *g*) and the supernatant was taken to measure the UV–Vis absorption at 410 nm. pH conditions:1 mL Mn/QD SAC ([Mn] = 10 ng/µL) and CAT (1 U/mL) were dispersed in 1 × PBS with the pH of 4, 7, 8, and 11 respectively for 48 h. 20 mM H_2_O_2_ was added to reaction for 1 h. Titanium sulfate colorimetry was used to detect residual H_2_O_2_. 20 µL reaction solution was added into 1 mL titanium sulfate solution, the mixture was centrifuged for 20 min (6740 *g*) and the supernatant was taken to measure the UV–Vis absorption at 410 nm.

### Cell culture

HT22 (CC-Y2137) and bEnd.3 (CC-Y2019) cells were obtained from China Center for Type Culture Collection. Cell line used was morphologically confirmed according to the information provided by culture collections. HT22 and bEnd.3 cells were both pre-cultured with DMEM medium containing 10% fetal bovine serum (FBS), 60 μg/mL penicillin, and 100 μg/mL streptomycin sulfate, in a constant temperature (37 °C) incubator with 5% CO_2_ and humid atmosphere. All the cell lines presented in this study were tested for mycoplasma contamination and they were free of mycoplasma contamination.

### Cytotoxicity test

HT22 and bEnd.3 cells were planted in 96-well plates (5 × 10^4^ cells per well) and cultured in an incubator containing 5% CO_2_ and humid gas at 37 °C for 24 h. Then the culture medium was replaced by fresh DMEM medium containing different concentrations of Mn/QD SAC (0, 25, 50, 100, 250, and 500 μg/mL) for co-incubation for 24 h. After washing twice gently with 1 × PBS buffer, 200 µL DMEM containing 10 µL WST-8 were added for another 20 min incubation, finally the absorbance at 450 nm was measured by the enzyme microplate reader, and the cell viability was calculated according to the absorbance.

### Clearance of intracellular ROS

HT22 cells were planted in a 6-well cell culture plate with a density of 1 × 10^5^ cells per well for 24 h, and then incubated with 1×PBS buffer containing 30% FBS and 20 mM H_2_O_2_ for 10 min to create the high ROS environment. Different concentrations of Mn/QD SAC ([Mn] = 0.25, 0.50, 1.25, and 2.50 ng/µL) were also added for co-incubation. Then the culture dish was gently cleaned twice with 1×PBS buffer, and 1 μL DCFH-DA was added to incubate for another 20 min, after washing twice gently with 1 × PBS buffer, and finally observed under a fluorescence microscope.

### Protection against intracellular ROS to nerve cells

The protective effect of Mn/QD SAC on nerve cells against ROS was evaluated by two methods.

Live-dead cell staining: Firstly, Live-Dead Cell Staining Kit was used to investigate the protection against ROS. 1 × 10^5^ HT22 cells were planted in a 6-well cell culture plate for 24 h, then the cells were treated with DMEM containing 600 µM H_2_O_2_, Mn/QD SAC ([Mn] = 1.5 ng/µL) were also added for co-incubation for 24 h. After washing twice gently with 1 × PBS buffer, Calcein-AM and PI were added for another 20 min incubation, finally observed under a fluorescence microscope and obtained images with NIS-Elements v.F 4.0.

CCK-8 Kit Assay: The protective effect against ROS of Mn/QD SAC was also investigated with CCK-8 Kit Assay. HT22 cells were planted in 96-well plates (5 × 10^4^ cells per well) and cultured in an incubator containing 5% CO_2_ and humid gas at 37 °C for 24 h. Then the culture medium was replaced by fresh DMEM medium containing 600 µM H_2_O_2_, different concentrations of Mn/QD SAC ([Mn] = 0.30, 0.75, and 1.50 ng/µL) were also added for co-incubation for 24 h. After washing twice gently with 1×PBS buffer, 200 µL DMEM containing 10 µL WST-8 were added for another 20 min incubation, finally the absorbance at 450 nm was measured by the enzyme microplate reader, and the cell viability was calculated according to the absorbance.

### Animal handing

For in vivo experiments, male and female mice with identical numbers were used. BALB/c mice aged ~8 weeks were purchased from Hubei Provincial Academy of Preventive Medicine. All mice were housed under specific pathogen–free (SPF) conditions (temperature ~22 °C, humidity ~50%) with a 12/12 h dark/light cycle and had free access to food (purchased from Xie tong Organism, SWS9102) and water throughout the study. The mice were anesthetized with isoflurane before experimental procedures, such as TBI model construction and imaging. After the experiment, the mice were euthanized by cervical dislocation, and all efforts were made to minimize pains.

### Weight-drop (WD) TBI models

The WD TBI model with moderate or severe injury was constructed according to the published procedures^52,68^. In short, brain contusion was caused by the free fall impact. First, the heads of healthy BALB/c mice (aged ~8 weeks) were carefully shaved, and after isoflurane anesthesia, they were fixed on the stereotaxic apparatus, and a metal rod (weighted about 65 g) were placed 8 cm above the head of the mice. Then release a free fall from a fixed slide, and the tip of the metal rod (diameter = 3 mm) impacted on the right hemisphere of the mice. Finally, the mice were placed on a 30 °C insulation blanket till they fully awakened.

### Hemolytic test

5% rabbit red blood cells (purchased from Meryer, Cat M52775) were incubated with 0.9% NaCl (negative control), ultra-pure water (positive control), and Mn/QD SAC (0.4 mg/mL, the concentration in blood when imaging in vivo) in an incubator for 15 min or 24 h. After centrifugation (3 min, 160 *g*), the supernatant was taken to measure the absorbance at 540 nm and calculate the proportion of hemolysis. Since the Mn/QD SAC itself has absorption at 540 nm, the same concentration of Mn/QD SAC was used as the control for comparison.

### Dynamic and high-resolution cerebrovascular imaging of TBI in NIR-IIb window

First, TBI mouse models were constructed by the above-mentioned method. About 3 h later, the mice were anesthetized with isoflurane and placed on the NIR-II small animal imaging system. The dynamic and high-resolution cerebrovascular imaging of TBI mice were performed with the magnification setup (3.3× or 10×). Then set the imaging parameters and start to collect video images, at the same time, 200 µL Mn/QD SAC (3.72 mg/mL) were injected through the tail vein to observe the dynamic blood perfusion in the left and right brains of the TBI mice. The video images were exported from the LightField software (Princeton Instruments) provided by imaging system. In this experiment, the healthy mice were used as a control at the same conditions. The specific imaging parameters were as follows: The excitation light source was an 808 nm laser at the power density of 100 mW/cm^2^, exposure time was 150 ms and 980 nm LP plus 1500 nm LP emission filters were used for NIR-IIb fluorescent channels.

### Brain immunofluorescence, immunohistochemistry and western blot analysis

First, TBI mouse models were constructed by the above-mentioned method. About 3 h later, 200 µL Mn/QD SAC (3.7 mg/mL), PEGylated Ag_2_Te (3.7 mg/mL) or 1×PBS buffer were injected through the tail vein of the TBI mice. In this experiment, healthy mice were taken as negative control. The mice were euthanized at different time points (12 h, 24 h, or 10 days post TBI) and gather their brains to soak in 4% paraformaldehyde. After 3 days, the brain tissues were embedded in paraffin and cut into slices.

For immunofluorescence, the slices were stained by the following procedures: Firstly, the slices were dewaxed in xylene, dehydrated in alcohol at different concentration gradients, and then sealed with 3% hydrogen peroxide for 10 min. According to the method in the manual to repair the antibody, then added the primary antibody mixture to co-incubate at 37 ^o^C for 2 h, washed them with 1 × PBS for 3 times. For primary antibody, anti-Iba1 (1:500, Servicebio, GB11105), anti-MMP-9 (1:500, Servicebio, GB11132), anti-α-SMA (1:300, Servicebio, GB111364), anti-Aqp4 (1:600, Servicebio, GB11529), anti-IgG (1:500, Servicebio, GB25301), anti-ZO-1 (1:200, Servicebio, GB111981), anti-Neun (1:200, Servicebio, GB11138) and anti-BrdU (1:100, Servicebio, GB12051) were used to stain the brain slices. After that, added the second antibody mixture and washed them with 1×PBS for 3 times after incubation, stained the nucleus with DAPI and sealed with anti-fluorescence quencher. The immunofluorescence staining images were obtained on PANNORAMIC MIDI digital slide scanner equipped with 10× objective (3DHISTECH, Hungary) with Pannoramic Scanner v.3.0.3 (Pannoramic Scanner software). The fluorescence images were analyzed using CaseViewer v. 2.4 and QuantCenter v.2.2 (3DHISTECH, Hungary). The ROI were selected near the injury site on the right hemisphere of brains. 3 animals per group with single slice per animal were evaluated for the semi-quantification of immunofluorescence staining.

For immunohistochemistry, the slices were stained by the following procedures: Firstly, the sections were placed in a 60 °C oven for dewaxing, the endogenous peroxidase was blocked with hydrogen peroxide, and then the sections were soaked in 0.01 M sodium citrate buffer solution for antigen repair. After washing with 1×PBS buffer for 3 times, use primary antibody, anti-IL-1β (1:200, Servicebio, GB12113), anti-IL-6 (1:200, Servicebio, GB11117) and anti-TNF-α (1:400, Servicebio, GB11188) to incubate with slices, after that, added the second antibody mixture and washed them with 1×PBS for 3 times after incubation. Then the slices were treated with 3,3N-Diaminobenzidine Tertrahydrochloride (DBA) and H_2_O_2_ for color development, and finally dehydrated with ethanol gradient, and sealed with xylene and neutral gum.

Western blot analysis was performed as previously described^20^. Brain tissues harvested from mice with different treatment were lysed with RIPA buffer (Beyotime, Cat P0013B). The protein concentrations were normalized by a BCA protein kit (Beyotime, Cat P0012S). In brief, the protein was isolated by SDS-PAGE using a wet electron imprinting system (Bio-Rad) and transferred to a PVDF membrane (Roche). The membrane was sealed in skim milk (5%) for 1 h and incubated with the primary antibody overnight at 4 °C. Antibodies against the following proteins were used for western blot: anti-VEGF (1:500, Servicebio, GB11034). The uncropped and unprocessed scans of the blots were provided in the Source Data file.

### ELISA for inflammatory cytokine

First, TBI mouse models were constructed by the above-mentioned method. About 3 h later, 200 µL Mn/QD SAC (3.7 mg/mL) or 1×PBS buffer were injected through the tail vein of the TBI mice. In this experiment, healthy mice were taken as negative control. The mice were euthanized at 12 h post TBI and gather their brains rapidly. The brain tissues were ground on the ice and centrifuged at 4 °C for 10 min (11,400 *g*). Supernatant protein concentration was measured with bicinchoninic acid method and used for inflammation-related cytokine quantification. ELISA kit for IL-1β (Beyotime, Cat PI301), IL-6 (Beyotime, Cat P326) and TNF-α (Beyotime, Cat PT512) were used to detect inflammation levels according to the instructions provided by the manufacturer.

### Animal exercise ability test and neurological score

Firstly, 30 BALB/c mice (aged ~8 weeks) were randomly divided into 3 groups (10 per group). Random numbers were generated using the “RAND() function” in Microsoft Excel. The mice were trained (balance beam, climbing rod and suspension rope experiment) for one week (2 days apart each time, 3 training times in total), and the neural scoring rules were formulated according to the test results. Then the mice were treated with different treatments: (I) TBI + PBS, (II) TBI + Mn/QD SAC, (III) TBI + Ag_2_Te. The motor ability was tested respectively at 4, 8, and 15 days after treatments, and the comprehensive neurological scores were obtained according to the scoring rules in Supplementary Tables 2–4. Each test counts for one third of the total neurological score. For each mouse, two investigators were involved as follow: The first investigator administered the treatments based on the randomization table. This investigator was the only person aware of the treatment group (Mn/QD SAC) allocation. A second investigator (unaware of treatment group) was responsible for the animal exercise ability tests and assessed neurological scores.

### Biodistribution investigation

18 healthy BALB/c mice (aged ~8 weeks) were randomly divided into 3 groups (6 per group) and injected with 200 µL Mn/QD SAC through tail vein. The mice were killed at 2 h, 8 h, and 168 h after injection respectively, and their main organs (the heart, liver, spleen, lungs, kidneys, intestine, stomach, and brain) were taken out for ex vivo tissues imaging on NIR-II imaging system.

### Biosafety investigation

12 healthy BALB/c mice (aged ~8 weeks) were randomly divided into 2 groups (6 per group). Then the mice were treated with different treatments: (I) PBS, (II) Mn/QD SAC. After different treatments, the weight of mice was carefully recorded every two days for 30 days. On the 30-th day, the main organs (brain, heart, liver, spleen, lungs, and kidneys) of mice were taken out for H&E staining.

### Statistics and reproducibility

Data are represented as the mean ± standard deviation (SD). Two-tailed two-sample Student’s *t* test was used for two-group comparisons. All statistical tests were performed using OriginPro v. 2018 (OriginPro Software) and GraphPad Prism software v. 8.0 (GraphPad Software). In all types of statistical analysis values of *P* < 0.05 were considered significant. The results of cellular fluorescence imaging, in vivo fluorescence imaging, immunofluorescence staining, immunohistochemistry staining, TEM imaging and H&E staining were repeated independently more than 3 times with similar results.

### Reporting summary

Further information on research design is available in the Nature Portfolio Reporting Summary linked to this article.

## Supplementary information

Supplementary Information

Description of Additional Supplementary Information

Supplementary Movie 1

Supplementary Movie 2

Supplementary Movie 3

Supplementary Movie 4

Supplementary Movie 5

Reporting Summary

## Data Availability

The data generated in this study are available within the Article, Supplementary Information or Source Data file. Source data are provided with this paper.

## Source data

Source Data
