# Supplementary material for: Near-infrared-IIb emitting single-atom catalyst for imaging-guided therapy of blood-brain barrier breakdown after traumatic brain injury
Source: Nat Commun. 2023 Jan 13;14:197. doi: 10.1038/s41467-023-35868-8 (PMC9839749; doi:10.1038/s41467-023-35868-8)
Supplement: Supplementary file 1 — Supplementary Information [file 41467_2023_35868_MOESM1_ESM.pdf]

Supplementary Information for "Near-Infrared-IIb Emitting Single-Atom Catalyst for Imaging-Guided Therapy of Blood-Brain Barrier Breakdown after Traumatic Brain Injury"

Biao Huang[1], Tao Tang[2], Shi-Hui Chen[1], Hao Li[3], Zhi-Jun Sun[3,*], Zhi-Lin Zhang[1,*], Mingxi Zhang[2,*], Ran Cui[1,*]

[1]College of Chemistry and Molecular Sciences, Wuhan University, 430072 Wuhan, China.

[2]State Key Laboratory of Advanced Technology for Materials Synthesis and Processing, Wuhan University of Technology, 430070 Wuhan, China.

[3]The State Key Laboratory Breeding Base of Basic Science of Stomatology (Hubei-MOST) & Key Laboratory of Oral Biomedicine Ministry of Education, School & Hospital of Stomatology, Wuhan University, 430079 Wuhan, China.

[*]Email: sunzj@whu.edu.cn; zlzhang@whu.edu.cn; mxzhang@whut.edu.cn; cuiran@whu.edu.cn

The PDF file includes:

Supplementary Figures 1-18
Supplementary Tables 1-5

a

Feed molar ratios of Mn:Ag	Mn content in Mn/QD SAC (wt%)
12.5%	0.22±0.04
15%	0.27±0.03
20%	0.34±0.02

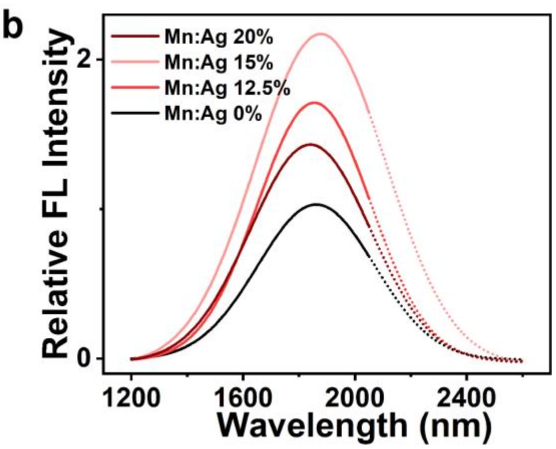

Supplementary Fig. 1 Fluorescence optimization with Mn:Ag feed ratio. a ICP-AES results of Mn/QD SAC with different doping amount of Mn synthesized under different feed molar ratios of Mn:Ag. Data are presented as mean ± SD (n = 3 independent samples). **b** Fluorescence spectrum of Mn/QD SAC synthesized under different Mn:Ag feed molar ratios. When Mn:Ag feed molar ratios was 15%, Mn/QD SAC displayed the highest fluorescence intensity. Source data are provided as a Source Data file.

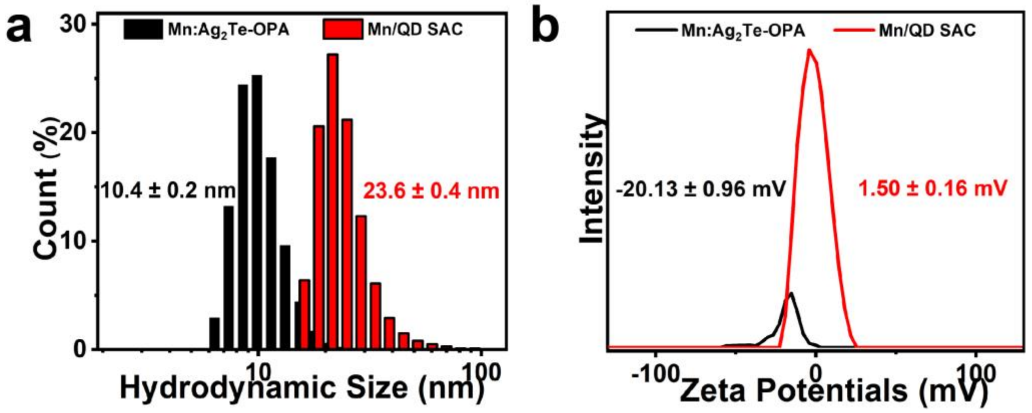

Supplementary Fig. 2 Hydrodynamic size and zeta potentials characterization of Mn/QD SAC. a Hydrodynamic size distributions of OPA-modified Mn-doped Ag_2Te QDs (Mn:Ag_2Te-OPA) and Mn/QD SAC. **b** Zeta potentials of Mn:Ag_2Te-OPA and Mn/QD SAC. All Data are presented as mean ± SD (*n* = 3 independent samples). Source data are provided as a Source Data file.

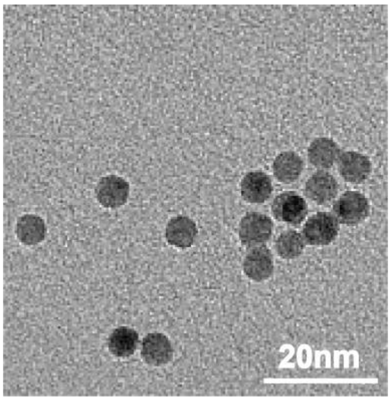

Supplementary Fig. 3 TEM image of Mn/QD SAC in 1×PBS buffer.

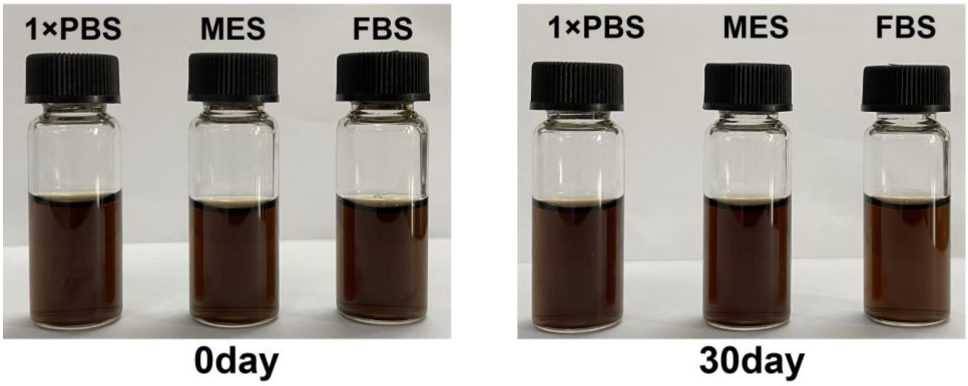

Supplementary Fig. 4 Storage stability of Mn/QD SAC in different solutions (1×PBS buffer, MES buffer and FBS).

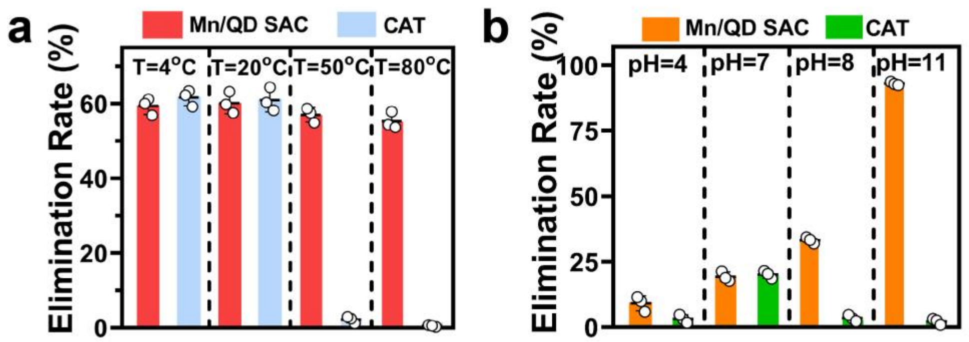

Supplementary Fig. 5 Catalytic activity stability investigation. a H_2O_2 elimination rate of Mn/QD SAC and CAT under different temperature conditions (4, 20, 50, and 80 ºC). **b** H_2O_2 elimination rate of Mn/QD SAC and CAT under different pH conditions (pH=4, 7, 8, and 11). All data are presented as mean ± SD (*n* = 3 independent samples). Source data are provided as a Source Data file.

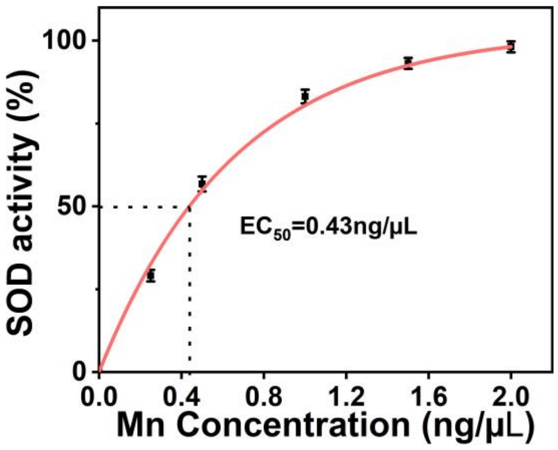

Supplementary Fig. 6 SOD activity versus different concentrations of Mn/QD SAC. Data are presented as mean ± SD (n = 3 independent samples). Source data are provided as a Source Data file.

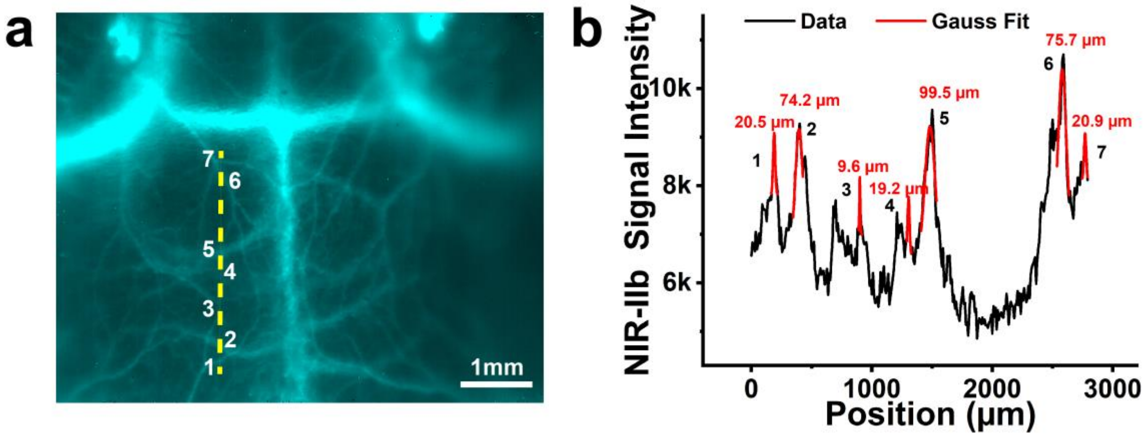

Supplementary Fig. 7 In vivo high-resolution NIR-IIb imaging for the brain of BALB/c mice. **a** High-resolution NIR-IIb image of brain with a 10× magnification setup. **b** NIR-IIb intensity profiles of the brain along yellow dashed lines in panel (a). The Gauss fitting was conducted to obtain the apparent width of cerebral micro-vessels numbered 1 to 7 and shown in panel (a). Source data are provided as a Source Data file.

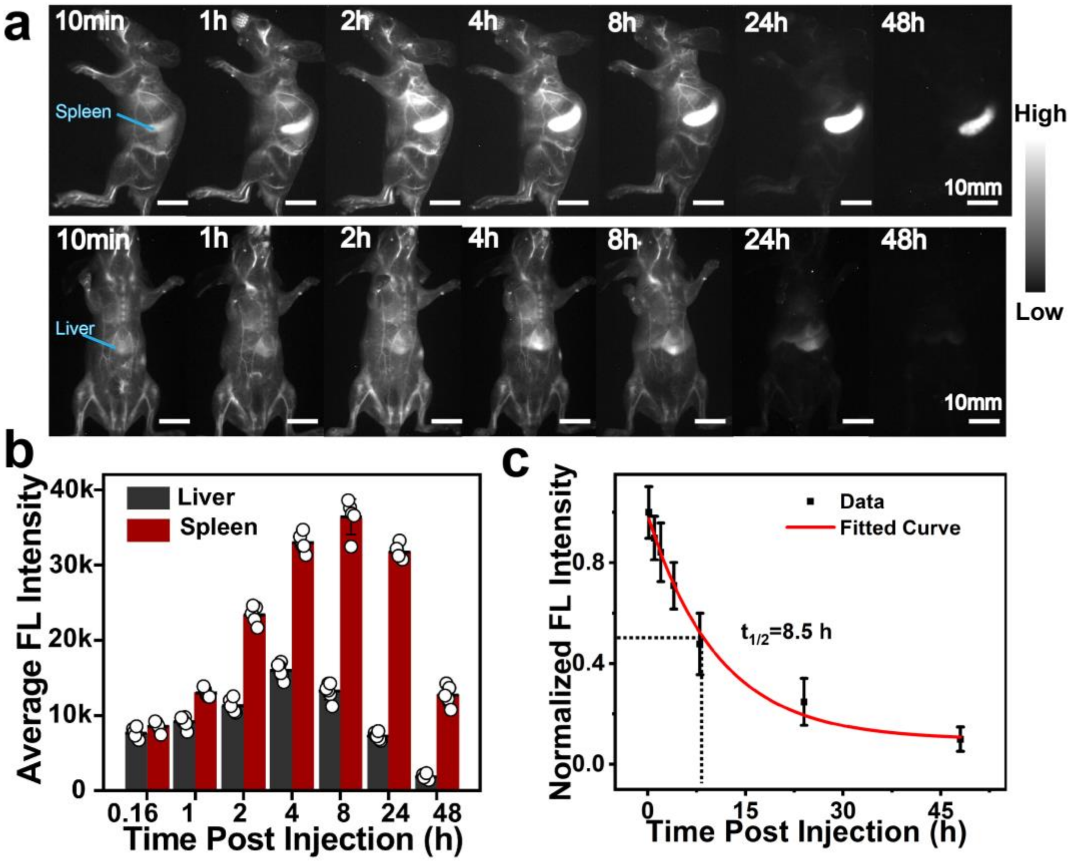

Supplementary Fig. 8 In vivo noninvasive NIR-IIb imaging for the whole body of BALB/c mice. **a** NIR-IIb imaging of mice in whole body with lateral (upper) and supine position (down). **b** Fluorescence intensity changes of mouse spleen and liver in vivo over time. Data are presented as mean ± SD (n = 6 independent samples). **c** Fitting curve for the half-life of Mn/QD SAC in vivo according to the fluorescence semi-quantitative analysis. Data are presented as mean ± SD (n = 6 independent samples). Source data are provided as a Source Data file.

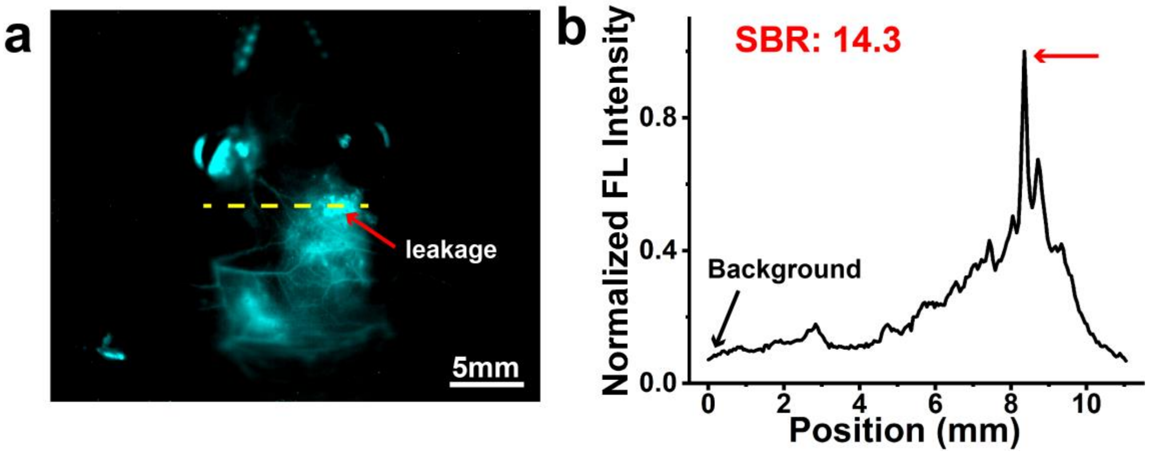

Supplementary Fig. 9 In vivo high-resolution NIR-IIb imaging for the brain of BALB/c mice post TBI. **a** NIR-IIb image of brain in TBI mouse. **b** Normalized NIR-IIb intensity profiles of the brain along yellow dashed lines in panel (a). The background signal was pointed by the black arrow. The highest intensity of leakage was pointed out by the red arrow (corresponding to the red arrow in panel a). The SBR was calculated to be 14.3. Source data are provided as a Source Data file.

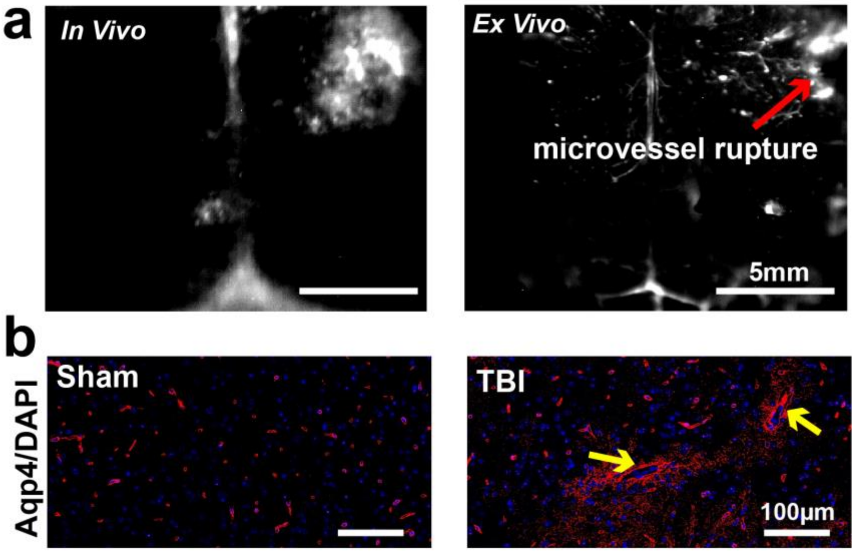

Supplementary Fig. 10 Pathological evidence of BBB breakdown in TBI mouse brain. **a** In vivo and *ex vivo* NIR-IIb imaging for the TBI mouse brain. The red arrow points to the location of the cerebral microvascular rupture. **b** Representative immunostaining images of the brain sections of sham or TBI mice with anti-Aqp4 for aquaporin-4 (Red). The nucleus of cells were stained with DAPI (Blue). The yellow arrow points to the location of over-expression of aquaporin-4.

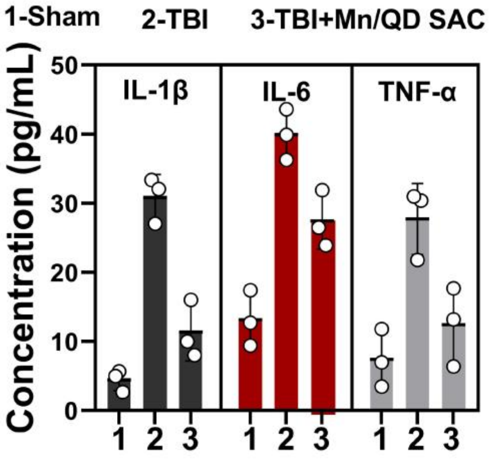

Supplementary Fig. 11 The expression level of three pro-inflammatory factors (IL-1β, IL-6, and TNF-α) in the brain of mice with different treatments by ELISA. Data are presented as mean ± SD (n = 3 independent samples). Source data are provided as a Source Data file.

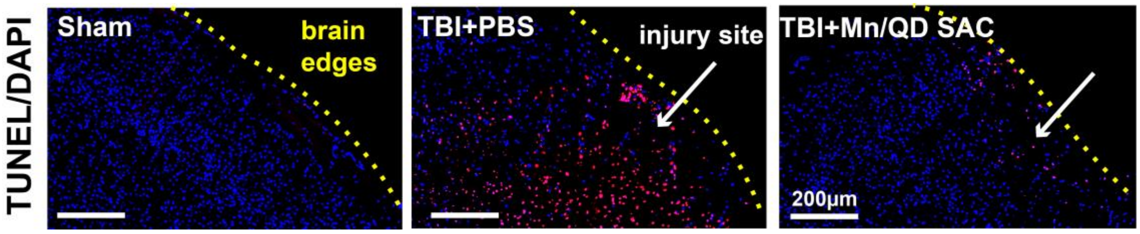

Supplementary Fig. 12 Apoptotic population in brain tissues at 24 h after TBI with TUNEL method. The yellow dashed lines outline the edges of brains. The white arrows point to the injury site of brains. The nucleus of cells were stained with DAPI (Blue), and the signal of cell apoptosis was labeled with TUNEL (Red).

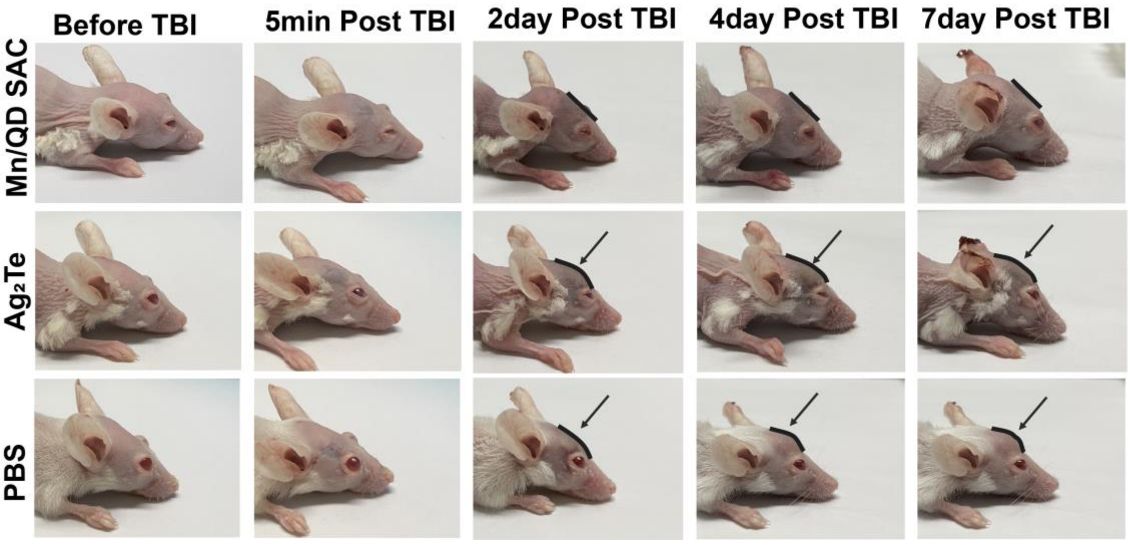

Supplementary Fig. 13 Digital photos of brain changes with time in TBI mice after different treatments. Black lines or curves outline the mouse brain in the side view. The black arrow indicates the presence of brain edema.

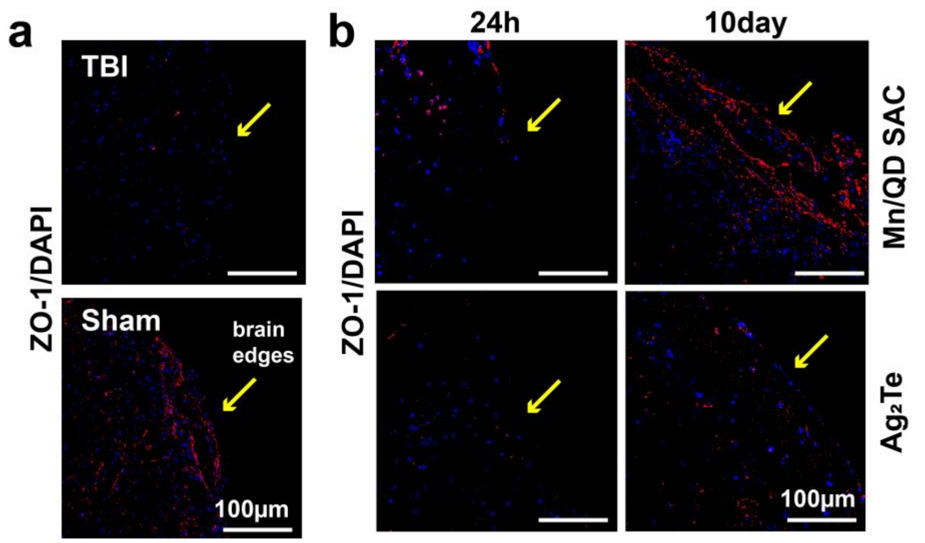

Supplementary Fig. 14 Expression of tight junction protein in BBB after treatment. **a** Representative immunostaining images of the brain sections of sham or TBI mice with anti-ZO1. **b** Representative immunostaining images of the brain sections obtained at 24 h or 10-th day post TBI with anti-ZO-1 for ZO-1 (Red). The nucleus of cells were stained with DAPI (Blue). The yellow arrows point to the edges of brains. After treatment with Mn/QD SAC, the expression of ZO-1 in the brain edges of mice upgraded significantly, indicating the recovery of BBB.

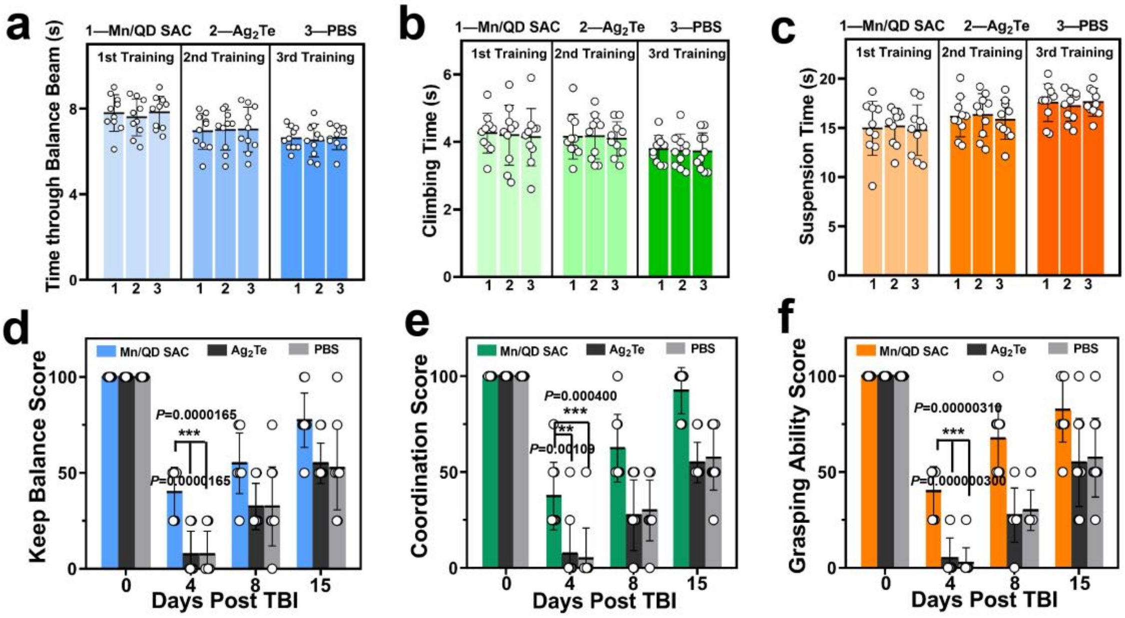

Supplementary Fig. 15 Animal behavior testy for neurological scoring. **a-c** Training performance of healthy mice in balance beam experiment (a), climbing rod experiment (b), and suspension rope experiment (c). **d-f** Motor ability scores of TBI mice in balance beam test (d), climbing rod test (e) and suspension rope test. (f) during recovery period. All data are presented as mean ± SD (*n* = 10 independent samples). Statistical significance was calculated via two-tailed two-sample Student's t test (d-f). (**) P < 0.01; (***) P < 0.001. Source data are provided as a Source Data file.

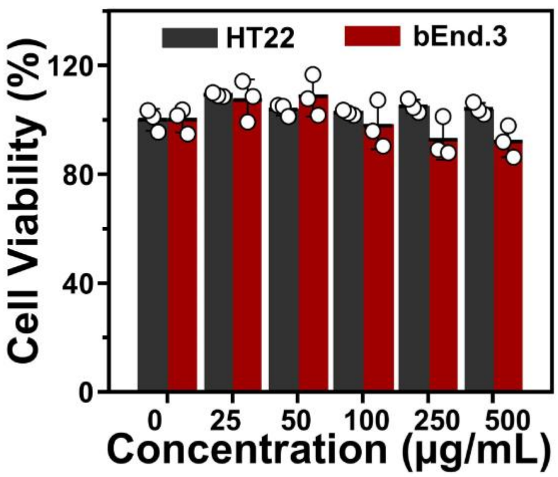

Supplementary Fig. 16 Cell viabilities of HT22 and bEnd.3 cells incubated with Mn/QD SAC for 24 hours at various concentrations (0, 25, 50, 100, 250, and 500 µg/mL). Data are presented as mean ± SD (*n* = 3 independent samples). Source data are provided as a Source Data file.

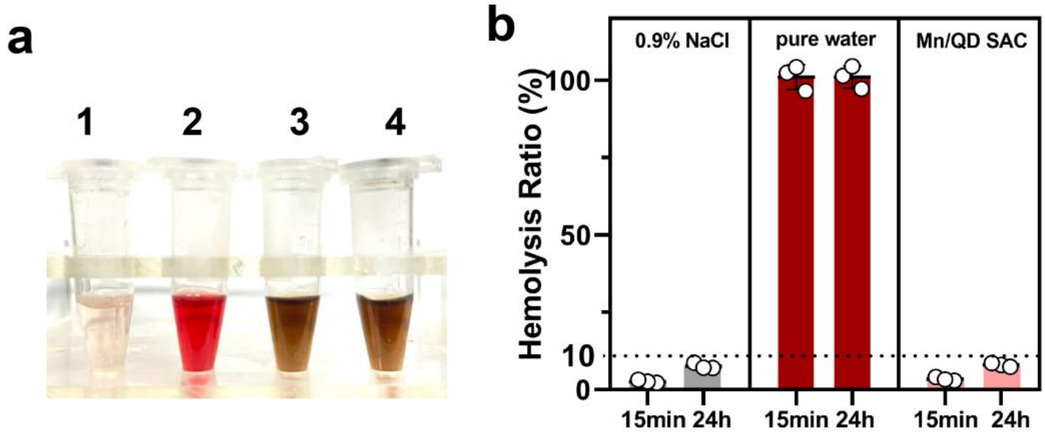

Supplementary Fig. 17 Hemolytic test for Mn/QD SAC. a The image of supernatant of red blood cells after incubating with 0.9% NaCl (1, negative control), ultra-pure water (2, positive control) and 0.4 mg/mL Mn/QD SAC (3) for 24 h. 4 represents the pure Mn/QD SAC (0.4 mg/mL). **b** The ratio of hemolysis after incubation with 0.9% NaCl, ultra-pure water, and 0.4 mg/mL Mn/QD SAC for 15 min or 24 h. Data are presented as mean ± SD (*n* = 3 independent samples). Source data are provided as a Source Data file.

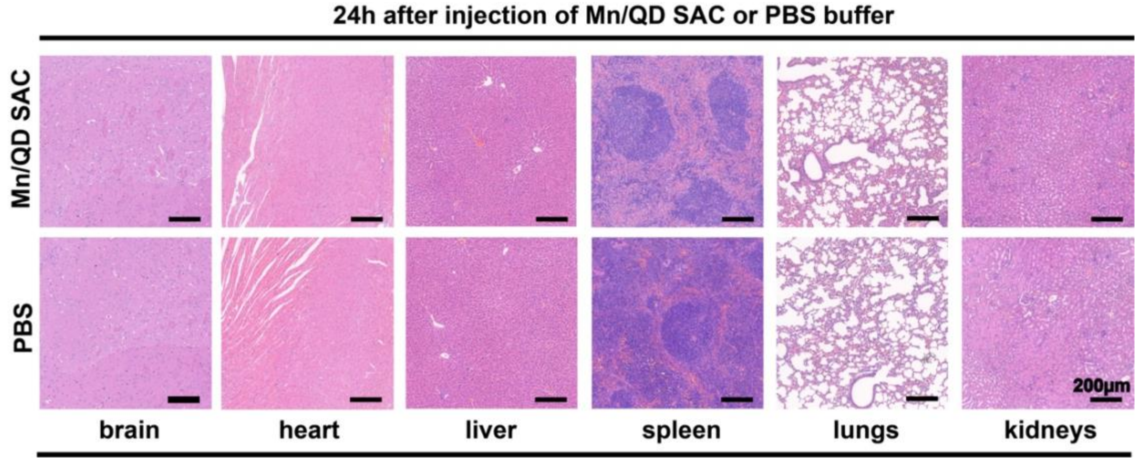

Supplementary Fig. 18 Representative H&E staining images of the major organs (brain, heart, liver, spleen, lungs, and kidneys) of the mice at 24 h after injection of Mn/QD SAC.

Element	Ag			Te			Mn		
Mass concentration (mg/mL)	8.96	7.43	4.57	4.10	3.54	2.16	0.03	0.03	0.02
wt%	68.44	67.54	67.70	31.32	32.18	32.00	0.24	0.28	0.30

Supplementary Table 1 The element content of each element of Mn/QD SAC was determined by ICP-AES.

Score description	Score
Fall from the balance beam or move less than 5 cm within 30 s	0
Keep on the balance beam, and the moving distance is 5-15 cm within 30 s	25
Keep on the balance beam, and the moving distance is 15-30 cm within 30s	50
Keep on the balance beam, and the moving distance is 30-40 cm within 30 s	75
Keep on the balance beam, and the moving distance is 5-15 cm within 30 s	100

Supplementary Table 2 Scoring rules in balance beam experiment.

Score description	Score
It is difficult to grasp the climbing rod and fall from the rod	0
Be able to grasp the lever and climb from top to bottom within 60 s	25
Be able to grasp the lever and climb from top to bottom within 30 s	50
Be able to grasp the lever and climb from top to bottom within 10 s	75
Be able to grasp the lever and climb from top to bottom within 5 s	100

Supplementary Table 3 Scoring rules in climbing pole experiment.

**Score description**	**Score**
Unable to grasp the suspension rope and fall down within 4 s	0
Be able to grasp the suspension rope and hold it for 4-8 s	25
Be able to grasp the suspension rope and hold it for 8-12 s	50
Be able to grasp the suspension rope and hold it for 12-16 s	75
Be able to grasp the suspension rope and hold it over 16 s	100

Supplementary Table 4 Scoring rules in suspension rope experiment.

Biochemical indicators	Test value	Normal range of value
ALT	42.69 ± 3.76 U/L	10.06-96.47 U/L
AST	197.89 ± 33.39 U/L	36.31-235.48 U/L
ALP	47.50 ± 12.81 U/L	22.52-474.35 U/L
BUN	21.37 ± 2.73 mg/dL	10.81-34.74 mg/dL
CREA	27.82 ± 8.52 µmol/L	10.91-85.09 µmol/L

Supplementary Table 5 The serum biochemical indicators of mice at 24 h after injection of Mn/QD SAC. Data are presented as mean ± SD (*n* = 3 independent samples).
