# Supplementary material for: Near-infrared-IIb emitting single-atom catalyst for imaging-guided therapy of blood-brain barrier breakdown after traumatic brain injury
Source: Nat Commun. 2023 Jan 13;14:197. doi: 10.1038/s41467-023-35868-8 (PMC9839749; doi:10.1038/s41467-023-35868-8)
Supplement: Supplementary file 2 — Description of Additional Supplementary Information [file 41467_2023_35868_MOESM2_ESM.docx]

Description of additional supplementary information

**Supplementary Movie 1:** Dynamic NIR-IIb imaging of brain with TBI mouse.

**Supplementary Movie 2:** Dynamic NIR-IIb imaging of brain with healthy mouse.

**Supplementary Movie 3:** Balance beam experiment tested at 8-day after treatment.

**Supplementary Movie 4:** Climbing rod experiment tested at 8-day after treatment.

**Supplementary Movie 5**: Suspension rope experiment tested at 8-day after treatment.
