# Supplementary material for: Near-infrared-IIb emitting single-atom catalyst for imaging-guided therapy of blood-brain barrier breakdown after traumatic brain injury
Source: Nat Commun. 2023 Jan 13;14:197. doi: 10.1038/s41467-023-35868-8 (PMC9839749; doi:10.1038/s41467-023-35868-8)
Supplement: Supplementary file 8 — Reporting Summary [file 41467_2023_35868_MOESM8_ESM.pdf]

nature portfolio

Corresponding author(s): Zhi-Jun Sun, Zhi-Lin Zhang, Mingxi Zhang, Ran Cui

Last updated by author(s): Dec 7, 2022

Reporting Summary

Nature Portfolio wishes to improve the reproducibility of the work that we publish. This form provides structure for consistency and transparency in reporting. For further information on Nature Portfolio policies, see our Editorial Policies and the Editorial Policy Checklist.

Statistics

For all statistical analyses, confirm that the following items are present in the figure legend, table legend, main text, or Methods section.

n/a	Confirmed	
☐	☒	The exact sample size (n) for each experimental group/condition, given as a discrete number and unit of measurement
☐	☒	A statement on whether measurements were taken from distinct samples or whether the same sample was measured repeatedly
☐	☒	The statistical test(s) used AND whether they are one- or two-sided<br>*Only common tests should be described solely by name; describe more complex techniques in the Methods section.*
☐	☒	A description of all covariates tested
☐	☒	A description of any assumptions or corrections, such as tests of normality and adjustment for multiple comparisons
☐	☒	A full description of the statistical parameters including central tendency (e.g. means) or other basic estimates (e.g. regression coefficient) AND variation (e.g. standard deviation) or associated estimates of uncertainty (e.g. confidence intervals)
☐	☒	For null hypothesis testing, the test statistic (e.g. F, t, r) with confidence intervals, effect sizes, degrees of freedom and P value noted<br>*Give P values as exact values whenever suitable.*
☒	☐	For Bayesian analysis, information on the choice of priors and Markov chain Monte Carlo settings
☒	☐	For hierarchical and complex designs, identification of the appropriate level for tests and full reporting of outcomes
☒	☐	Estimates of effect sizes (e.g. Cohen's d, Pearson's r), indicating how they were calculated

Our web collection on statistics for biologists contains articles on many of the points above.

Software and code

Policy information about availability of computer code

Data collection	NIS-Elements v.F 4.0, Pannoramic Scanner v.3.0.3, UVProbe v.2.33, FluorEssence v. 3.8, LightField software v. 6.4 (Princeton Instruments).
Data analysis	Softwares used for analysis include OriginPro v. 2018, GraphPad Prism v. 8.0 (GraphPad Software), Image-Pro Plus v. 6.0 (Media Cybernetics), LightField software v. 6.4 (Princeton Instruments), Excel 2016 (Microsoft), CaseViewer v. 2.4 (3DHISTECH),QuantCenter v.2.2(3DHISTECH).

For manuscripts utilizing custom algorithms or software that are central to the research but not yet described in published literature, software must be made available to editors and reviewers. We strongly encourage code deposition in a community repository (e.g. GitHub). See the Nature Portfolio guidelines for submitting code & software for further information.

Data

Policy information about availability of data

All manuscripts must include a data availability statement. This statement should provide the following information, where applicable:

- Accession codes, unique identifiers, or web links for publicly available datasets
- A description of any restrictions on data availability
- For clinical datasets or third party data, please ensure that the statement adheres to our policy

The data generated in this study within the Article and Supplementary Information are available and provided in the Source Data file. Source data are provided with this paper.

Human research participants

Policy information about studies involving human research participants and Sex and Gender in Research.

Reporting on sex and gender	N/A
Population characteristics	N/A
Recruitment	N/A
Ethics oversight	N/A

Note that full information on the approval of the study protocol must also be provided in the manuscript.

Field-specific reporting

Please select the one below that is the best fit for your research. If you are not sure, read the appropriate sections before making your selection.

[x] Life sciences [ ] Behavioural & social sciences [ ] Ecological, evolutionary & environmental sciences

For a reference copy of the document with all sections, see nature.com/documents/nr-reporting-summary-flat.pdf

Life sciences study design

All studies must disclose on these points even when the disclosure is negative.

Sample size	Although no sample size calculation was performed, sample sizes for the in vivo experiments are similar to those generally employed and accepted in the field (Nat. Commun. 8, 737 (2017).; Sci. Adv. 6, eabb2695 (2020).;ACS Nano 13, 11552-11560 (2019).) and were sufficient to support our conclusions with statistical significance. Sample sizes for the in vitro experiments are also based on previous work (Nat. Commun. 11, 2788 (2020), Sci. Adv. 7, eabk1210 (2021)).
Data exclusions	No data were excluded.
Replication	Experiments were repeated independently at least three experiments with similar results. All experiments were reproduced to reliably support conclusions stated in the manuscript.
Randomization	Our samples/mice were allocated randomly. Random numbers were generated using the RAND() function in Microsoft Excel.
Blinding	The investigators were blinded to group allocation during data collection and analysis. Due to the obvious wounds on the brains and abnormal behavioral activities, the investigators could not be blinded to whether the mice had TBI or sham treatment. Instead, single-blind experiments were performed for the groups of TBI mice injected with PBS buffer, Ag2Te, or Mn/QD SAC. For each mouse, two investigators were involved as follow: the first investigator administered the treatments based on the randomization table. This investigator was the only person aware of the treatment group (Mn/QD SAC) allocation. A second investigator (unaware of treatment group) was responsible for the animal exercise ability tests and assessed neurological scores.

Reporting for specific materials, systems and methods

We require information from authors about some types of materials, experimental systems and methods used in many studies. Here, indicate whether each material, system or method listed is relevant to your study. If you are not sure if a list item applies to your research, read the appropriate section before selecting a response.

Materials & experimental systems

n/a	Involved in the study	
[ ]	[x]	Antibodies
[ ]	[x]	Eukaryotic cell lines
[x]	[ ]	Palaeontology and archaeology
[ ]	[x]	Animals and other organisms
[x]	[ ]	Clinical data
[x]	[ ]	Dual use research of concern

Methods

n/a	Involved in the study	
[x]	[ ]	ChIP-seq
[x]	[ ]	Flow cytometry
[x]	[ ]	MRI-based neuroimaging

Antibodies

Antibodies used

The following primary antibodies were used for immunofluorescence. They are listed as antigen first, followed by supplier, catalog number as applicable.
1) anti-MMP-9, 1:500,Servicebio, GB11132;
2) anti-α-SMA, 1:300, Servicebio, GB111364;
3) anti-Aqp4, 1:600, Servicebio, GB11529;
4) anti-IgG, 1:500, Servicebio, GB25301;
5) anti-ZO-1, 1:200, Servicebio, GB111981;
6) anti-Neun, 1:200, Servicebio, GB11138;
7) anti-BrdU,1:100, Servicebio, GB12051;
8) anti-Iba1,1:500, Servicebio, GB11105.

The following primary antibodies were used for immunohistochemistry. They are listed as antigen first, followed by supplier, catalog number as applicable.
1) anti-IL-1β, 1:200, Servicebio, GB12113;
2) anti-IL-6, 1:200, Servicebio, GB11117;
3) anti-TNF-α, 1:400, Servicebio, GB11188.

The following primary antibodies were used for western blotting. They are listed as antigen first, followed by supplier, catalog number as applicable.
1) anti-VEGF, 1:500, Servicebio, GB13034.

Validation

No customized antibodies were used. Validation data of the antibodies purchased from commercial vendors are available on the manufactures' website and datasheets.

1) anti-MMP-9
https://www.servicebio.com/goodsdetail?id=1401
2) anti-α-SMA
https://www.servicebio.com/goodsdetail?id=3743
3) anti-Aqp4
https://www.servicebio.com/goodsdetail?id=1268
4) anti-IgG
https://www.servicebio.com/goodsdetail?id=272
5) anti-ZO-1
https://www.servicebio.com/goodsdetail?id=4916
6) anti-Neun
https://www.servicebio.com/goodsdetail?id=1406
7) anti-BrdU
https://www.servicebio.com/goodsdetail?id=591
8) anti-IL-1β
https://www.servicebio.com/goodsdetail?id=606
9) anti-IL-6
https://www.servicebio.com/goodsdetail?id=1393
10) anti-TNF-α
https://www.servicebio.com/goodsdetail?id=4760
11) anti-VEGF
https://www.servicebio.com/goodsdetail?id=1334
12)anti-Iba1
https://www.servicebio.com/goodsdetail?id=1385

Eukaryotic cell lines

Policy information about cell lines and Sex and Gender in Research

Cell line source(s)

Mouse hippocampal neuronal cells (HT22) and mouse brain microvascular endothelial cells (bEnd.3) were obtained from China Center for Type Culture Collection.

Authentication

Cell line used was morphologically confirmed according to the information provided by culture collections.

Mycoplasma contamination

All the cell lines presented in this study were tested for mycoplasma contamination and they were free of mycoplasma contamination.

Commonly misidentified lines
(See ICLAC register)

No commonly misidentified cell lines were used.

Animals and other research organisms

Policy information about studies involving animals; ARRIVE guidelines recommended for reporting animal research, and Sex and Gender in Research

Laboratory animals	For in vivo experiments, male and female mice with identical numbers were used. BALB/c (~8 weeks) were purchased from Hubei Provincial Academy of Preventive Medicine. All mice were housed under specific pathogen–free (SPF) conditions (temperature ~22 ° C, humidity ~50%) with a 12/12 h dark/light cycle and had free access to food (purchased from Xie tong Organism, SWS9102) and water throughout the study.The mice were anesthetized with isoflurane before experimental procedures, such as TBI model construction and imaging. After the experiment, the mice were euthanized by cervical dislocation, and all efforts were made to minimize pains.
Wild animals	The study did not involve wild animals.
Reporting on sex	Identical numbers of male and female mice were used in this study and no significant sex difference was observed.
Field-collected samples	The study did not involve samples collected from field.
Ethics oversight	Ethical approval of this study was obtained from the Animal Ethics Committee of the School and Stomatology of Wuhan University. All animal experimental procedures were performed in accordance with the Regulations for the Administration of Affairs Concerning Experimental Animals approved by the State Council of the People's Republic of China.

Note that full information on the approval of the study protocol must also be provided in the manuscript.
